# Physiologically-Based Pharmacokinetic (PBPK) Modeling Providing Insights into Fentanyl Pharmacokinetics in Adults and Pediatric Patients

**DOI:** 10.3390/pharmaceutics12100908

**Published:** 2020-09-23

**Authors:** Lukas Kovar, Andreas Weber, Michael Zemlin, Yvonne Kohl, Robert Bals, Bernd Meibohm, Dominik Selzer, Thorsten Lehr

**Affiliations:** 1 Department of Clinical Pharmacy, Saarland University, 66123 Saarbrücken, Germany; lukas.kovar@uni-saarland.de (L.K.); andimattweber@gmail.com (A.W.); dominik.selzer@uni-saarland.de (D.S.); 2 Department of General Pediatrics and Neonatology, Saarland University Medical Center, 66421 Homburg, Germany; michael.zemlin@uks.eu; 3 Fraunhofer Institute for Biomedical Engineering IBMT, 66280 Sulzbach, Germany; yvonne.kohl@ibmt.fraunhofer.de; 4 Department of Internal Medicine V, Saarland University, 66421 Homburg, Germany; robert.bals@uks.eu; 5 Department of Pharmaceutical Sciences, College of Pharmacy, The University of Tennessee Health Science Center, Memphis, TN 38163, USA; bmeibohm@uthsc.edu

**Keywords:** physiologically-based pharmacokinetic (PBPK) modeling, fentanyl, neonates, norfentanyl, pediatric scaling, drug–drug interaction (DDI), pharmacokinetics

## Abstract

Fentanyl is widely used for analgesia, sedation, and anesthesia both in adult and pediatric populations. Yet, only few pharmacokinetic studies of fentanyl in pediatrics exist as conducting clinical trials in this population is especially challenging. Physiologically-based pharmacokinetic (PBPK) modeling is a mechanistic approach to explore drug pharmacokinetics and allows extrapolation from adult to pediatric populations based on age-related physiological differences. The aim of this study was to develop a PBPK model of fentanyl and norfentanyl for both adult and pediatric populations. The adult PBPK model was established in PK-Sim^®^ using data from 16 clinical studies and was scaled to several pediatric subpopulations. ~93% of the predicted AUC_last_ values in adults and ~88% in pediatrics were within 2-fold of the corresponding value observed. The adult PBPK model predicted a fraction of fentanyl dose metabolized to norfentanyl of ~33% and a fraction excreted in urine of ~7%. In addition, the pediatric PBPK model was used to simulate differences in peak plasma concentrations after bolus injections and short infusions. The novel PBPK models could be helpful to further investigate fentanyl pharmacokinetics in both adult and pediatric populations.

## 1. Introduction

Fentanyl is a strong opioid—approximately 50- to 100-fold more potent compared to morphine—and is extensively used in the therapeutic fields of analgesia, sedation, and anesthesia both in adult and pediatric patients [1,2,3]. While clinical trials on the pharmacokinetics (PK) of fentanyl suggest several factors such as liver function impacting the dose–exposure relationship, the wide interindividual variability is still not completely understood [3]. As fentanyl is a substrate of the cytochrome P450 (CYP) iso-enzyme 3A4, fentanyl PK can be altered by concomitant administration of CYP3A4 inhibitors and inducers (drug–drug interactions, DDIs) [3].

The major route of metabolic clearance was assumed to be mediated via CYP3A4 metabolizing fentanyl to the inactive metabolite norfentanyl [3,4]. However, recent research activities have suggested a strong involvement of additional metabolic pathways and hypothesized unknown metabolites [5,6].

In critically ill neonates, analgesic therapy is commonly administered, since pain can act as a stressor increasing mortality in this population [1]. Indeed, fentanyl is the opioid analgesic most frequently used in neonatal intensive care units [1], which highlights the importance of fentanyl in pediatrics. Yet, there is still a lack of knowledge regarding fentanyl PK in children [2]. The desired analgesic and sedating effects resulting from administration of fentanyl usually lead to an improvement of respiratory compliance [7,8]. However, adverse drug reactions (ADRs), such as bradycardia, respiratory depression, and, in rare cases, chest wall rigidity, might occur even after low doses of fentanyl administration [1,8]. A recent meta-analysis by Ziesenitz and colleagues concluded the need for further research on fentanyl, especially in larger cohorts and special subpopulations such as preterm neonates and children with hepatic or renal impairment [2]. However, pediatric PK studies are difficult to conduct and are often impeded by ethical and logistic challenges, many of which are unique to pediatrics [9].

Physiologically-based pharmacokinetic (PBPK) modeling can be used for evaluating and extending existing knowledge on drug disposition derived from in vitro and in vivo investigations into unstudied subpopulations and clinical scenarios [10,11]. An increasing number of drug applications submitted to the US Food and Drug Administration (FDA) and the European Medicines Agency (EMA) have investigated the impact of hepatic disease, pharmacogenomics, and DDIs on drug PK with the help of PBPK modeling [10,12]. Previous PBPK efforts on fentanyl have focused on methodological aspects of simplifying PBPK models [13], on a PBPK approach to support the development of Provisional Advisory Levels (PALs) for hazardous agents [14], and on simulating thyroid and testes tissue concentrations [15], respectively.

In pediatrics, PBPK approaches have also proven its usefulness in designing and optimizing clinical trials and are supported by both the FDA and the EMA [12,16,17,18,19,20]. For a priori PBPK predictions in pediatrics, the PBPK model first needs to be informed and evaluated with published PK data in adults and subsequently extrapolated to pediatric populations—a workflow which has recently been implemented and successfully executed for several drugs [10,21,22,23,24,25].

The aim of the presented work was to develop a whole-body parent-metabolite intravenous PBPK model of fentanyl and norfentanyl in adults as a foundation for further assessment of fentanyl PK and to extrapolate the adult PBPK model for the prediction of plasma concentration–time profiles as well as individual clearance parameters in pediatric patients. The novel PBPK models are publicly available in the Open Systems Pharmacology (OSP) repository as clinical research tools to support the design of clinical trials in specific populations as well as the development of novel drug formulations. The Supplementary Materials serve as an additional reference manual including detailed model performance evaluation.

## 2. Materials and Methods

### 2.1. Software

The PBPK models were developed with the PK-Sim^®^ modeling software (version 9.0, 2020, www.open-systems-pharmacology.org), which is part of the OSP Suite [11]. Clinical plasma data from scientific literature was digitized using GetData Graph Digitizer version 2.26.0.20 (S. Fedorov, 2013) according to best practices [26]. Model input parameters were optimized using the Monte Carlo algorithm implemented in PK-Sim^®^. PK parameter and model performance measure calculation as well as graph plotting were performed with the R programming language version 3.6.1 (R Foundation for Statistical Computing, Vienna, Austria, 2019) and R Studio^®^ version 1.2.5019 (R Studio, Inc., Boston, MA, USA, 2019).

### 2.2. PBPK Model Building for Adult Populations

The adult fentanyl parent-metabolite model building was initiated with an extensive literature search to obtain information on physicochemical properties as well as distribution, metabolism and excretion processes of fentanyl and norfentanyl. The gathered information was utilized to implement relevant drug–protein interactions (i.e., for transport proteins and enzymes) and to inform drug-dependent model input parameters. Published clinical studies of intravenous fentanyl administration in single- and multiple-dose regimens were used to extract plasma profiles and measured fractions of fentanyl dose excreted unchanged in urine. The fentanyl and norfentanyl plasma profiles were divided into an internal training and an external test dataset. Studies for the training dataset were selected containing a wide range of profiles (i.e., different dosing regimens, timing and frequency of sampling, measurements of norfentanyl, measurements of arterial and venous plasma concentrations, etc.). The test dataset was used for model evaluation.

Distribution and elimination processes including CYP enzymes as well as drug transporters were implemented according to the literature [5,27,28]. For the fentanyl model, these included (1) metabolism of fentanyl to the inactive metabolite norfentanyl via CYP3A4 and CYP3A7, (2) an unspecific hepatic pathway metabolizing fentanyl to other non-specified metabolites, (3) distribution and excretion via P-glycoprotein (P-gp), and (4) renal excretion through glomerular filtration. It should be noted that the actual role of CYP3A7 in the metabolism of fentanyl is suspected but the true nature of involvement still remains unknown [27,29]. Since CYP3A4 and CYP3A7 exhibit a similar substrate spectrum and CYP3A7 is the major fetal form of CYP3A [30], CYP3A7 might be important for PK predictions of fentanyl in pediatric populations and was therefore incorporated in the model. Considering that norfentanyl is predominantly eliminated via urine, renal clearance was implemented and estimated during the parameter optimization step [5]. Tissue expression distribution of the metabolizing enzymes and P-gp in all model compartments was implemented according to the PK-Sim^®^ expression database [31]. Model input parameters, which could not be adequately obtained from literature, were estimated by fitting the parent-metabolite model to the training dataset. For detailed information on PBPK model building see Section 1 in the Supplementary Materials.

### 2.3. PBPK Modeling in Pediatrics 

For a priori predictions of plasma concentration–time profiles as well as clearance parameters in pediatrics, the adult PBPK model was scaled to pediatric populations of different age groups. For this, the adult virtual populations were replaced by pediatric virtual populations, changing the physiological and anatomic parameters describing the human body. As a result, anatomic and physiological parameters as well as CYP3A4 and CYP3A7 tissue concentrations were scaled to the particular pediatric target population taking age-related changes such as size of tissue compartments and maturation of enzyme abundances into account. The used ontogeny functions for CYP3A4 and CYP3A7 are depicted in the PK-Sim^®^ ontogeny database [32]. Since ontogenetic information regarding the transport protein P-gp were not implemented in PK-Sim^®^, the age-dependent protein abundance was estimated via the published ontogeny function from Prasad et al. [33]. While changes in anatomy and physiology during liver maturation were considered, the rate constant of the implemented unspecific hepatic clearance process was assumed to be independent of age. The unbound fraction of fentanyl was scaled for each particular pediatric population using the method of McNamara and Alcorn for alpha-1-acid glycoprotein [34,35] and compared to a range of published literature values. The remaining drug-dependent parameters were fixed to the values of the adult PBPK model. Next, the extrapolated PBPK model was applied to predict plasma profiles in pediatric populations of different age ranges (i.e., preterm and full-term neonates, infants and children). Additionally, 65 individual clearance values from preterm and full-term neonates were predicted and compared to observed values from three clinical trials [36,37,38].

### 2.4. PBPK Model Evaluation and DDI Modeling

Predicted and observed plasma concentrations as well as predicted and observed areas under the plasma concentration–time curve from the first to the last data point (AUC_last_) were compared in goodness-of-fit plots for both the adult and the pediatric PBPK models. Moreover, all simulated fentanyl and norfentanyl plasma concentration–time trajectories were visually compared to the plasma profiles observed from the respective clinical trial. In order to visualize the interindividual variability of the model, virtual populations of 100 individuals were created covering the patient characteristics of the corresponding clinical study population. Detailed information on virtual populations can be found in Section 1.3 in the Supplementary Materials. For plasma profile simulation for individual patients, populations of 100 individuals with the same patient demographics were used, only allowing variability in the expression of the implemented enzymes, transporters and the unspecific hepatic clearance. Furthermore, two quantitative model performance measures were calculated for the adult and pediatric PBPK models: the mean relative deviation (MRD) of the predicted from the observed plasma concentrations for each plasma profile as well as the geometric mean fold error (GMFE) of the computed AUC_last_ ratios. The sensitivity of the PBPK models to single-parameter changes (local sensitivity analysis) was estimated with PK-Sim^®^. Detailed information on the calculation of MRD, GMFE and sensitivity analysis can be found in Section 3 in the Supplementary Materials. 

For further evaluation of the adult PBPK model, a DDI scenario of fentanyl and the CYP3A4 inhibitor voriconazole was predicted and compared with the plasma profiles observed in a clinical trial. For this, the developed fentanyl-norfentanyl PBPK model was coupled with a recently published PBPK model of voriconazole [39]. The mathematical implementation of the competitive and irreversible interaction is described in Section 2 in the Supplementary Materials.

For pediatrics, individual and population mean clearance values extracted from three clinical trials were compared to predictions from the pediatric PBPK model. Finally, for all models, the percentages of model-predicted AUC_last_ and clearance values falling within 2-fold of the corresponding observed values were calculated.

## 3. Results

### 3.1. PK Data for PBPK Model Development and Pediatric Scaling

For adult PBPK model development, 16 clinical studies including 24 different treatment arms were utilized, covering a broad dosing range of 0.3–60 µg/kg body weight intravenous fentanyl in single- and multiple-dosing regimens. Here, three treatment arms also reported plasma concentrations of the metabolite norfentanyl. The dataset included one DDI study with voriconazole as the perpetrator drug as well as 14 study arms with fentanyl administration before or during surgeries. Nine treatment arms measured fentanyl concentrations in arterial blood, 14 in venous blood and one covered both sampling sites. Moreover, three clinical trials provided information on fractions of fentanyl dose excreted unchanged in urine [5,40,41]. All plasma profiles were digitized and split into an internal training (*n* = 9 profiles) and an external test dataset (*n* = 18 profiles). An overview of the included clinical studies in adults including study characteristics, dosing regimens, and the assignments to training and test dataset is shown in Table 1.

For predictive performance evaluation of the extrapolated pediatric PBPK model, five clinical trials investigating fentanyl plasma concentrations in preterm neonates, full-term neonates, infants and young children with mean age ranging from 32 weeks of gestational age to approximately three years of chronological age were identified. Moreover, individual clearance values of 65 preterm and full-term neonates were extracted from three published studies. An overview of the clinical studies in pediatrics including study characteristics and dosing regimens is provided in Table 2.

### 3.2. Adult PBPK Model Building and Evaluation

**Figure 1:**
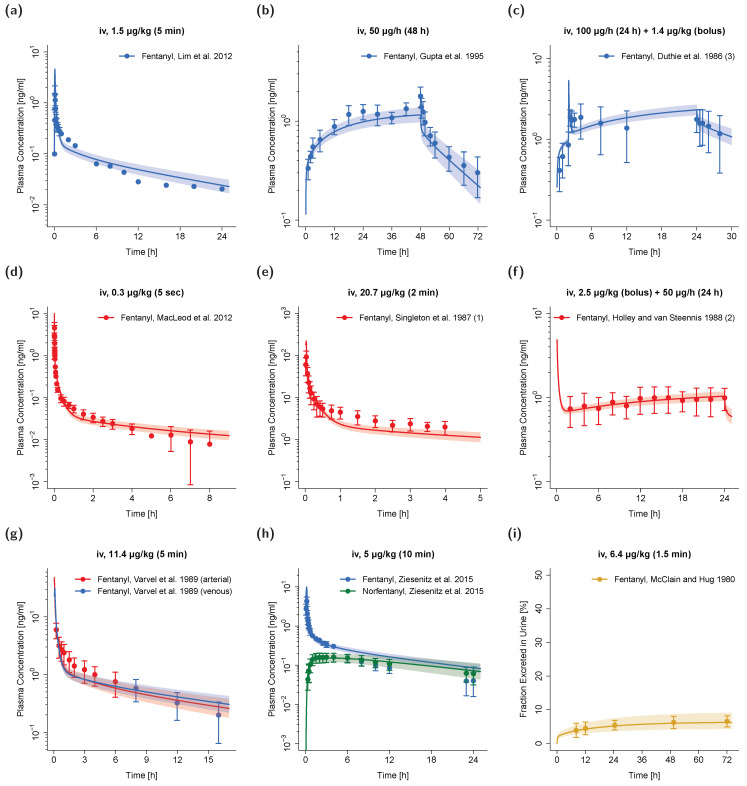
Fentanyl (blue: venous blood; red: arterial blood; orange: urine) and norfentanyl (green: venous blood) predicted and observed plasma concentration–time as well as fraction excreted in urine–time profiles after intravenous administration of fentanyl in adults. (**a**,**c**,**e**–**g**): selection of external test dataset; (**b**,**d**,**h**,**i**): selection of internal training dataset. Population simulations (*n* = 100) are shown as lines with shaded areas (geometric mean and geometric standard deviation). Observed data is shown as circles ± standard deviation if available. References with numbers in parentheses link to a specific observed dataset ID described in the study table (Table 1). Predicted and observed areas under the plasma concentration–time curve from the first to the last data point (AUC_last_) are compared in Table S5 of the Supplementary Materials. Predicted and observed plasma concentration–time profiles of all studies in adults (linear and semilogarithmic) are shown in Section 3.1 in the Supplementary Materials. iv: intravenous.

A whole-body adult PBPK model of fentanyl and its metabolite norfentanyl was built and comprehensively evaluated using arterial and venous plasma profiles as well as information on fraction of fentanyl dose excreted unchanged in urine. Model-predicted population profiles are compared to the corresponding study data in Figure 1 (selection of profiles from the training and test dataset) and in detail in Section 3.1 in the Supplementary Materials (all simulated studies, both on a semilogarithmic and linear scale). Simulated plasma profile trajectories of fentanyl for bolus/short-infusion administrations as well as long-term infusions are in close concordance with observed data. This holds true for both fentanyl venous and arterial blood plasma concentrations as well as for venous norfentanyl plasma concentrations.

**Figure 2:**
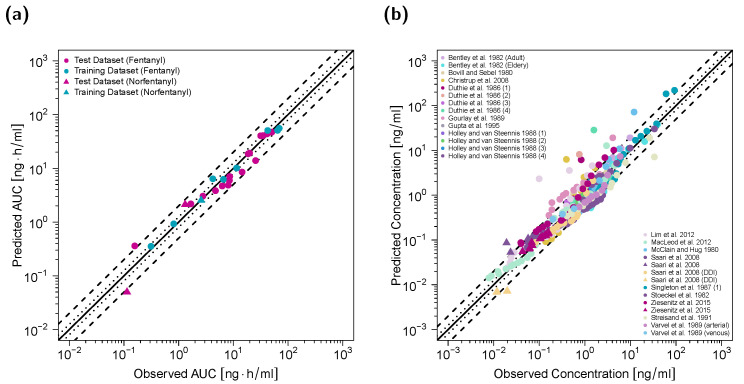
Predicted versus observed AUC_last_ values of fentanyl and norfentanyl grouped by test and training dataset (**a**) and predicted versus observed plasma concentrations (**b**) for the adult PBPK model. In (**a**), each symbol represents the AUC_last_ of a single plasma concentration–time profile (circles: fentanyl; triangles: norfentanyl). In (**b**), each symbol represents a single plasma concentration (circles: fentanyl; triangles: norfentanyl). The black solid lines mark the lines of identity. Black dotted lines indicate 1.25-fold, black dashed lines indicate 2-fold deviation. AUC_last_: area under the plasma concentration–time curve from the first to the last data point.

Goodness-of-fit plots of predicted versus observed AUC_last_ values and of predicted versus observed plasma concentrations are shown in Figure 2. Twenty-six out of 28 predicted AUC_last_ values (~93%) fell within the 2-fold acceptance criterion with an overall GMFE of 1.30. One AUC_last_ outside the 2-fold range was calculated for a venous fentanyl profile, which covered only the first 20 min after a fentanyl bolus administration [45]. The second outlier was calculated for a venous norfentanyl profile with observed norfentanyl concentrations close or below the lower limit of quantification (LLOQ) [6]. The MRD value for all plasma concentration simulations for the adult PBPK model was 1.77 with ~86% of all simulated plasma concentrations falling within 2-fold of the corresponding concentration observed. Detailed results on MRD values and AUC ratios calculated for all studies and results of the sensitivity analysis are presented in Sections 3.4–3.6 of the Supplementary Materials. 

**Figure 3:**
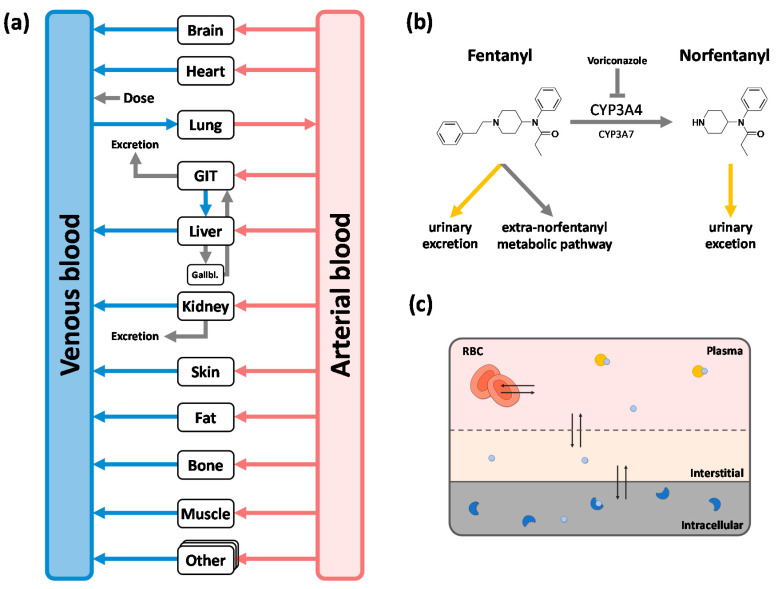
Structural overview of the whole-body physiologically-based pharmacokinetic (PBPK) model (**a**) and implemented elimination processes for fentanyl and norfentanyl (**b**). Each organ consists of a vascular space (containing plasma and red blood cells), interstitial space, and intracellular space. In (**a**,**c**), boxes indicate compartments, arrows denote in-/outflows, blue circles represent molecules, orange circles represent plasma proteins, and blue crescents denote enzymes. In (**b**), grey arrows indicate metabolic processes and yellow arrows indicate urinary excretion processes. CYP: cytochrome P450; GIT: gastrointestinal tract; Gallbl: gallbladder.

While the implemented unspecific hepatic clearance is responsible for approximately 60% of fentanyl elimination, the metabolism of fentanyl to norfentanyl via CYP3A4 and CYP3A7 covers approximately one-third of fentanyl elimination in the PBPK model. The urinary excretion is accountable for only a minor fraction of fentanyl elimination (~7%). Figure 3 shows a structural overview of the implemented elimination processes of fentanyl and norfentanyl as well as a structural overview of the PBPK model. Drug-dependent parameters of the final PBPK model are shown in Table 3. For detailed information including system-dependent model parameters, see Section 1 of the Supplementary Materials.

### 3.3. PBPK DDI Modeling

**Figure 4:**
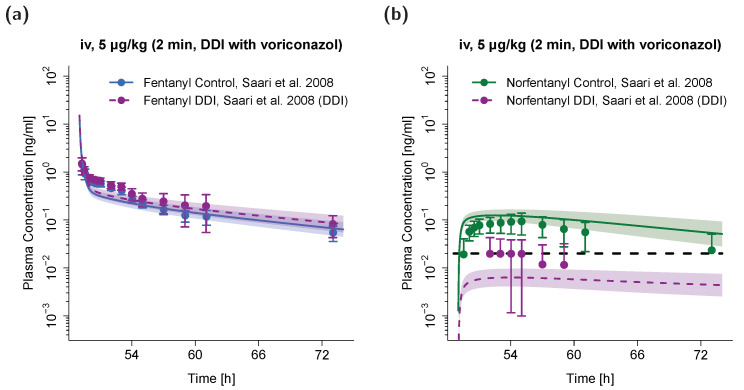
DDI scenario for fentanyl (**a**) and norfentanyl (**b**) with the perpetrator drug voriconazole in adults. Fentanyl and norfentanyl plasma concentrations during concomitant administration of voriconazole are shown in purple. Plasma concentrations during sole fentanyl administration are shown in blue (fentanyl) and green (norfentanyl), respectively. Population simulations (*n* = 100) are shown as lines with shaded areas (geometric mean and geometric standard deviation). Observed data is shown as filled circles ± standard deviation. References link to a specific observed dataset described in Table 1. Black dashed line depicts the specified lower limit of quantification (LLOQ) for norfentanyl [6]. Predicted and observed areas under the plasma concentration–time curve from the first to the last data point (AUC_last_) are compared in Table S5 of the Supplementary Materials. DDI, drug–drug interaction; iv, intravenous.

The adult PBPK model was used to predict a DDI scenario of fentanyl with concomitant administration of voriconazole. The simulated plasma concentration–time profiles are compared to the corresponding profiles observed from a clinical DDI study [6] in Figure 4. Here, a slight decrease in fentanyl AUC can be observed when simulating fentanyl administration with concomitant voriconazole compared to simulation of sole fentanyl administration. The corresponding predicted AUC_ratio_ (AUC_inhibition, predicted_/AUC_control, predicted_ = 1.22) and observed AUC_ratio_ (AUC_inhibition, observed_/AUC_control, observed_ = 1.33) were very similar. A strong relative decrease in norfentanyl AUC can be observed when simulating fentanyl administration with concomitant voriconazole compared to simulation of sole fentanyl administration. The predicted AUC_ratio_ for norfentanyl was 0.02 and the observed AUC_ratio_ 0.09. It should be noted that all observed plasma concentrations for norfentanyl during concomitant voriconazole administration were very close to or below the specified LLOQ (black dashed line in Figure 4b) [6].

### 3.4. Pediatric PBPK Model Building and Evaluation

The adult PBPK model was extrapolated to pediatric populations with mean ages ranging from 32 weeks gestational age in a preterm neonate population up to 2.9 years in a population of young children. In total, plasma concentration–time profiles and clearance values were predicted and compared to observed data for pediatric mean populations and individuals from five different clinical trials. 

**Figure 5:**
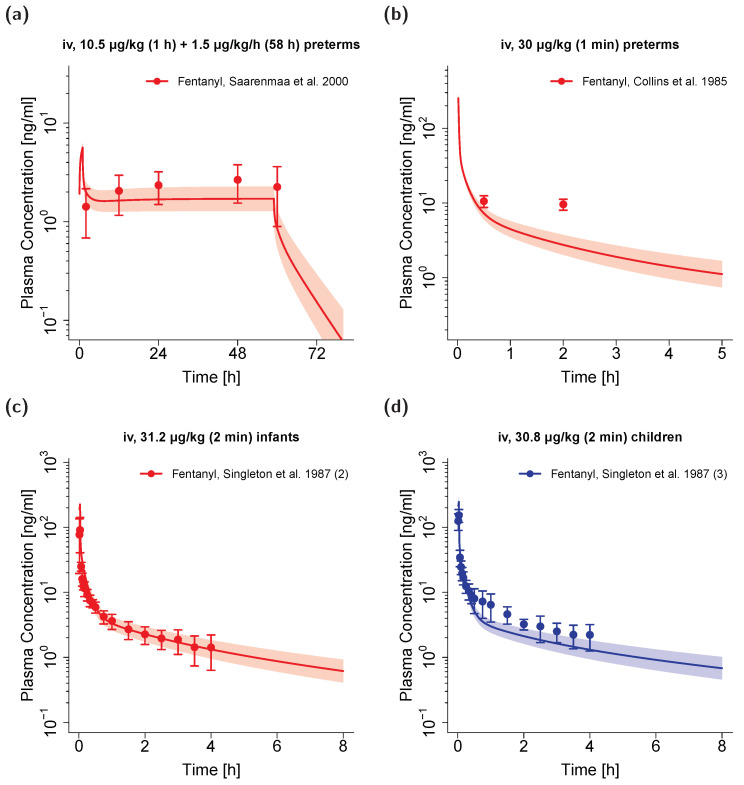
Fentanyl (red: arterial blood; blue: venous blood from central venous catheters) predicted and observed plasma concentration–time profiles after intravenous administration in preterm neonates (**a**,**b**), infants (**c**) and young children (**d**). Population simulations (*n* = 100) are shown as lines with shaded areas (geometric mean and geometric standard deviation). Observed data is shown as circles with standard deviation. References with numbers in parentheses link to a specific observed dataset ID described in Table 2. Predicted and observed area under the plasma concentration–time curve from the first to the last data point (AUC_last_) values are compared in Table S5 of the Supplementary Materials. Predicted and observed plasma concentration–time profiles of all studies in pediatrics are shown in Section 3.2 of the Supplementary Materials both on a linear and a semilogarithmic scale. iv: intravenous, preterms: preterm neonates.

The scaled unbound fraction of fentanyl resulted in values from 29% for the 2.9-year-old pediatric population to 33% in the preterm neonate populations. This range is in concordance with the measured unbound fentanyl fraction values in pediatrics from the literature (23–38%) [35,64,65]. All other drug-dependent parameters were fixed to the values of the adult PBPK model. Comparison of predicted and observed plasma concentration–time profiles are shown in Figure 5 (semilogarithmic, selection of plots) and in Section 3.2 of the Supplementary Materials (all plots, both linear and semilogarithmic).

**Figure 6:**
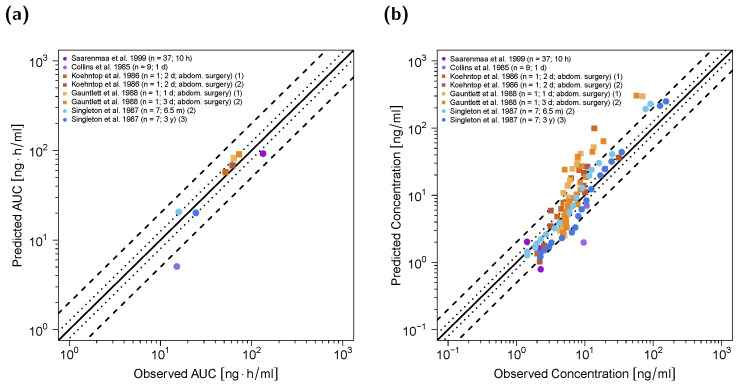
Predicted versus observed AUC_last_ (**a**) and plasma concentrations (**b**) of fentanyl for the pediatric PBPK model. Squares depict values for individual patients with adjusted clearances due to increased intraabdominal pressure as explained in Section 3.4; circles depict values for study populations without adjustment of clearances. In (**a**), each symbol represents the AUC_last_ of a single plasma concentration–time profile. In (**b**), each symbol represents a single plasma concentration. The black solid lines mark the lines of identity. Black dotted lines indicate 1.25-fold deviation; black dashed lines indicate 2-fold deviation. AUC_last_: area under the plasma concentration–time curve from the first to the last data point; abdom.: abdominal.

The predicted and observed plasma concentrations as well as AUC_last_ values are compared in goodness-of-fit plots in Figure 6, with 87.5% of AUC_last_ predictions located within the 2-fold range of the respective observed values and a GMFE of 1.38. Overall, MRD for plasma concentration predictions in pediatrics was calculated to be 2.03. A detailed overview of all MRD and GMFE values for pediatric predictions can be found in Tables S4 and S5 in the Supplementary Materials.

**Figure 7:**
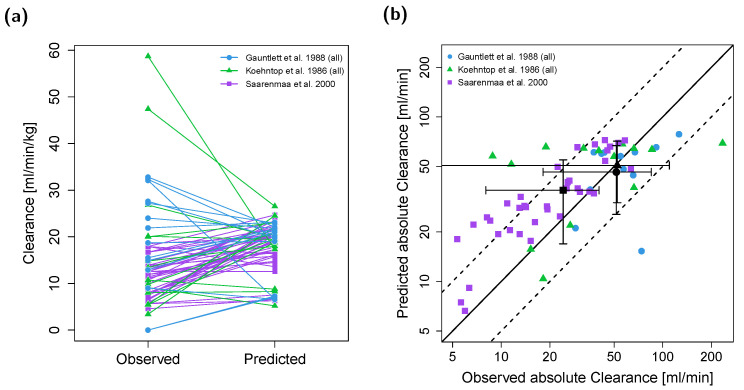
Predicted and observed clearance per bodyweight (**a**) as well as predicted versus observed absolute clearance values for the pediatric PBPK model (**b**). Each colored symbol represents the individual clearance of a patient; black symbols represent the mean clearances with standard deviations (circles refer to data from Gauntlett et al. [36]; triangles refer to data from Koehntop et al. [37]; squares refer to data from Saarenmaa et al. [38]). In (**b**), the black solid line marks the line of identity; black dashed lines indicate 2-fold deviation.

Individual clearance values were predicted and compared to the corresponding values observed from three clinical trials (see Table 2). Here, 72% of the predicted individual values (Figure 7a,b) and 100% of mean values (Figure 7b) are located within the 2-fold range of the corresponding observed values. 

### 3.5. Clearance in Neonates with Increased Intraabdominal Pressure

In studies by Gauntlett et al., Koehntop et al. and Saarenmaa et al., several neonates, who had abdominal surgery, showed a significantly reduced fentanyl clearance [36,37,38] and four corresponding individual plasma profiles were presented in the respective studies [36,37]. The authors hypothesized that the decreased clearance might be due to an increased intraabdominal pressure resulting in a decreased hepatic clearance [36,37,38]. Hence, the four observed plasma profiles were digitized and used to estimate decreased clearance values for the CYP3A4, CYP3A7 and unspecific hepatic clearance pathways for each individual. This resulted in a mean reduction in the metabolic clearance of ~83%. For detailed information see Section 1.2 of the Supplementary Materials. The resulting four individual plasma profiles are displayed in Figures S5 and S6 in the Supplementary Materials. 

## 4. Discussion

A whole-body PBPK model of fentanyl for adults has been built and evaluated by describing and predicting arterial (fentanyl) and venous (both fentanyl and norfentanyl) plasma concentration–time profiles as well as fractions of fentanyl dose excreted unchanged in urine following intravenous fentanyl administration. The utilized dataset comprised a wide dose range (0.3–60 µg/kg) including administrations ranging from an intravenous bolus to a 48-h continuous infusion. Following comprehensive evaluation, the adult PBPK model was applied to successfully predict a DDI scenario with voriconazole and was scaled to pediatric populations. Plasma concentration–time profiles and clearance parameters in pediatric patients were predicted with the extrapolated pediatric PBPK model and compared to observed data from five different clinical trials. The descriptive and predictive performance of the PBPK models has been demonstrated by (1) comparison of simulated to observed plasma profiles and clearance parameters, (2) the respective goodness-of-fit plots, (3) the calculation of MRD values as well as (4) the comparison of predicted to observed AUC_last_ values including the calculation of the respective GMFE. 

The adult PBPK model predicts the fraction of fentanyl dose metabolized to norfentanyl of ~33% and the fraction of fentanyl eliminated via an unspecific hepatic clearance of ~60% (hereinafter called “extra-norfentanyl metabolic pathway”). These findings are supported by the prediction results of the DDI study with the CYP3A4 inhibitor voriconazole as a perpetrator drug. Moreover, the fraction of fentanyl dose excreted unchanged in urine was calculated to be ~7%. While fraction excreted in urine is perfectly in accordance with the literature [5,40,41], reports about the fraction of fentanyl metabolized to norfentanyl are divergent. In vitro studies with liver microsomes from the 1990s suggested that metabolism to norfentanyl plays the major role in fentanyl elimination—mainly via CYP3A4 with little contribution of other CYP enzymes [4,27]. Yet, an in vivo DDI crossover study from 2015 showed that concomitant administration of the CYP3A4 and P-gp inhibitor ketoconazole significantly reduced norfentanyl AUC to 24% but increased fentanyl exposure by only ~33% [5]. This strong inhibition of norfentanyl production during ketoconazole treatment supports the assumption of a major involvement of CYP3A in norfentanyl formation [5]. However, Ziesenitz et al. calculated the metabolic clearance of fentanyl to norfentanyl to account only for ~23% of the systemic clearance and concluded that currently unknown metabolites exist [5]. With a fraction of fentanyl metabolized to norfentanyl of approximately one-third, our study supports the theory of an extra-norfentanyl metabolic pathway and currently unknown metabolites. The fact that ritonavir, a drug which interacts with numerous metabolizing enzymes and transporters, had a much more profound effect on fentanyl exposure (AUC increase by ~170%) than ketoconazole provides additional support for the involvement of other elimination pathways in addition to CYP3A4 [66]. It needs to be noted that, in this PBPK model, the extra-norfentanyl metabolic pathway was implemented as an unspecific clearance in the liver, but could also be located at various different sites. Further studies need to be conducted to investigate the characteristics of an extra-norfentanyl elimination pathway.

PBPK modeling permits rational scaling between adult and pediatric patients by defining the PK of a drug as a function of anatomy, physiology and biochemistry and successful applications have recently been shown in different modeling efforts [21,22,23]. This study demonstrates the applicability of PBPK modeling to predict both clearance values as well as plasma concentration–time profiles and the corresponding AUCs for the analgesic drug fentanyl for preterm neonates to up to 3-year-old children within a whole-body PBPK framework. Here, 87.5% of AUC_last_ predictions and 100% of predicted population mean clearances were within 2-fold of the respective values observed. The predicted to observed mean clearance ratios (CL_predicted_/CL_observed_) for the three included clinical trials were 0.97 and 0.90 for the full-term neonate populations and 1.48 for the preterm neonate population. However, as individual fentanyl PK is highly variable [1,37], prediction of individual plasma profiles and clearance values remains challenging. The model predicted 47 of 65 individual clearance values within 2-fold range based on information on weight and age (chronological and gestational if available). The presented ladder plot (Figure 7a) depicts noticeable mispredictions for some clearance values. This might be attributed to unknown individual CYP3A4, CYP3A7 and P-gp expressions as well as heterogeneity for the unbound fraction, which all exhibit high interindividual variability [32,33,35]. In the model, mean values for enzyme and transporter expressions as well as the unbound fraction were assumed for individual clearance simulations.

As it remains unknown which physiologic elimination pathways the unspecific hepatic clearance covers, no ontogeny information regarding this elimination process was available. Hence, it needs to be noted that the rate constant of this clearance process was not scaled in the model. While the overall reasonable predictive performance of the PBPK scaling supports this approach, the overpredicted clearance values as well as the underprediction of plasma profiles for the preterm neonate population (see Figure 5a,b) could indicate that the unspecific hepatic clearance is less pronounced in preterm neonates. Certainly, these effects could also be mediated by other factors such as the impact of concomitant comedication or the influence of surgery on the PK of fentanyl. 

Increased intraabdominal pressure can occur during abdominal surgery [37,67]. This might substantially decrease hepatic blood flow and eventually lead to a reduced fentanyl clearance [36,37,67]. In the PBPK model simulations, the clearance reduction for neonates with abdominal surgery and increased intraabdominal pressure (mean reduction in the metabolic clearance of ~83%) [36,37,38] showed an overall improvement of predictions. However, it should be noted that not all infants who had abdominal surgery showed a decreased clearance [36,37]. More research is required to further investigate the impact of abdominal surgery and intraabdominal pressure on the PK of fentanyl.

**Figure 8:**
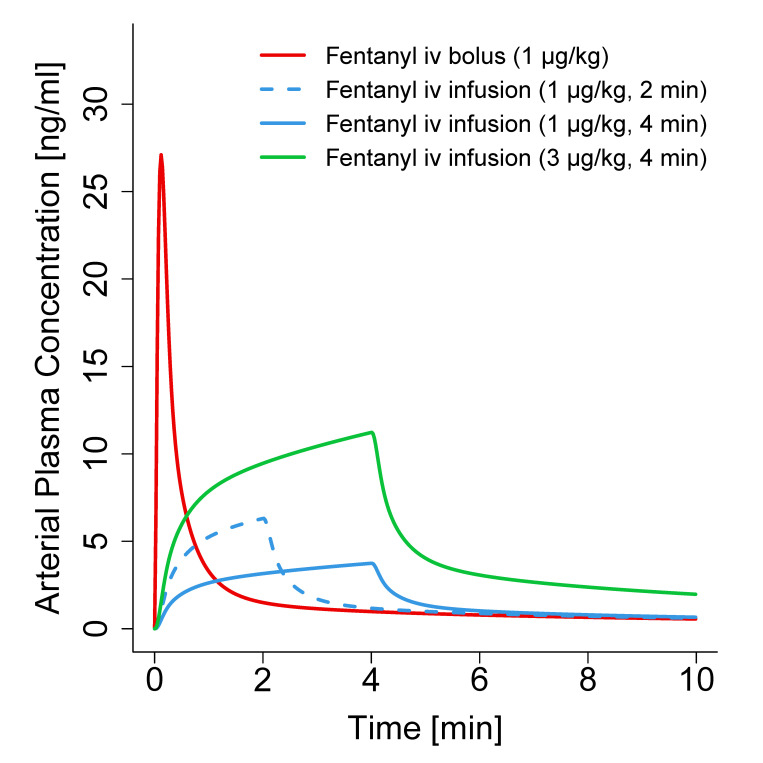
Simulations of arterial plasma concentration–time profiles after intravenous fentanyl bolus injection as well as 2- and 4-min infusions of different doses. Lines depict population geometric means (*n* = 100). iv: intravenous.

In most clinical scenarios, the administration of fentanyl leads to a desired analgesic and sedating effect, which, among others, usually results in an improvement of respiratory function [7,8]. However, in rare cases, fentanyl might cause a rigidity affecting the respiratory musculature, which can lead to chest wall rigidity—an ADR in pediatric as well as adult populations [1,8]. Albeit commonly assumed that chest wall rigidity occurs with rapid fentanyl administrations of high doses, Dewhirst et al. showed that the ADR may result from doses as low as 1 µg/kg, mostly from bolus injections lasting less than 15 s (15 of 21) [8]. Simulations with the developed PBPK model in neonates show a large difference in peak arterial plasma concentrations (Figure 8) after bolus injection (1 µg/kg, C_max_: 27.1 ng/mL), 2-and 4-min infusions (1 µg/kg, C_max_: 6.3 and 3.7 ng/mL, respectively), and even a 3 µg/kg 4 min infusion (C_max_: 11.2 ng/mL). 

These large differences of up to 7-fold peak concentrations might partly explain the more frequent occurrence of chest wall rigidity during bolus administration of fentanyl. However, the impact of the high interindividual variability [1,37] should not be disregarded, which might explain the occurrence of the ADR in other case reports with longer infusions [8].

Previous modeling efforts on fentanyl neither included the metabolite norfentanyl nor predicted DDI scenarios [13,14,15]. Consequently, the fraction of fentanyl metabolized to norfentanyl could not be assessed. Moreover, scaling the PBPK models to pediatric subpopulations in order to predict plasma profiles and clearance values was not part of the scope of the above-mentioned PBPK modeling studies.

Some limitations of the model should be discussed. As no information about the blood-to-plasma ratio in the different pediatric populations was available, the literature value of 0.87 was assumed to be age-independent. However, since the unbound fraction changes age-dependently [34,35,65], the blood-to-plasma ratio could also differ in pediatric compared to adult patients. This could be one of the reasons for some deviations when predicting the plasma concentration–time profiles.

Moreover, measurements of norfentanyl plasma concentrations were scarce and only available in clinical studies with adults for PBPK model building and evaluation [5,6]. Hence, PBPK model simulations for norfentanyl were only evaluated in this population and should be interpreted with these limitations in mind.

The impact of the metabolic process of CYP3A7 in our PBPK model on fentanyl AUCs was negligible, as shown in the local sensitivity analyses. Solely in PBPK model simulations for neonates, the CYP3A7 elimination process had a small impact. However, as no in vitro studies investigating CYP3A7 metabolism of fentanyl with information on the maximum reaction velocity and Michaelis–Menten constant were available, this process needs further investigation.

The ontogeny function for P-gp was adapted from a recent publication [33]. However, the ontogenetic information might need further evaluation since the peptides quantified by the used liquid chromatography–tandem mass spectrometry technique are not only formed from active P-gp but also by splice variants as well as non-glycosylated and truncated proteins [33,68,69].

Many of the PK samples in the analyzed studies were taken during different surgical procedures with largely varying co-medications (e.g., atropine, isoflurane, pancuronium, succinylcholine, and thiopental) possibly affecting fentanyl plasma concentrations. Consequently, some differences in model-predicted and observed plasma concentrations and clearance values could be a result of the varying study conditions.

In addition to intravenous administration, fentanyl is also administered via the transdermal, sublingual and nasal routes [3]. In particular, the continuous administration of fentanyl with transdermal patches is an important analgesic treatment in diseases with chronic pain [70]. Transdermal fentanyl treatment is approved for opioid-tolerant adult patients as well as opioid-tolerant children over two years of age [2,71]. As a sustained-release formulation, transdermal patches can potentially reduce plasma concentration fluctuations, ADRs such as constipation and the risk for non-adherence [70]. Based on the good model performance, the fentanyl PBPK models developed in this analysis could be augmented to mechanistically model and simulate the delivery of fentanyl via more complex formulations, such as transdermal or sublingual vehicles [72].

## 5. Conclusions

A whole-body PBPK model of fentanyl and its metabolite norfentanyl has been developed to predict fentanyl and norfentanyl arterial and venous plasma concentration–time profiles as well as fentanyl urinary excretion after intravenous administration in adults. The model was further evaluated by predicting a DDI scenario with the CYP3A4 inhibitor voriconazole. The fraction of fentanyl metabolized to norfentanyl of ~33% has been predicted, supporting the idea of an extra-norfentanyl metabolic pathway. Subsequently, the adult PBPK model has been successfully scaled to preterm and full-term neonate, infant as well as child subpopulations for predictions of plasma profiles and clearance parameters. With that, we add confidence to the potential of PBPK modeling to predict the PK in pediatric patients. The models are publicly available in the OSP repository. Thereby, the models contribute to a library of PBPK models for predictions in other DDI scenarios, could help to develop models for sustained release from complex formulations, and support future investigations of fentanyl and norfentanyl PK both in adult and pediatric populations.

## Figures and Tables

**Table 1:** Overview of clinical studies used for building and evaluation of the adult PBPK model of fentanyl and norfentanyl.

Clinical Study ID	Dose ^a^ [µg/kg]	Dose ^b^ [µg/h]	Administration	n	Female [%]	Age [Years]	Weight [kg]	Blood Sample	Surgery	Dataset	Reference
Bentley et al., 1982 (Adult)	10.0		iv (bolus)	5	100	36 ± 4	64 ± 3	arterial	yes	e	[42]
Bentley et al., 1982 (Eldery)	10.0		iv (bolus)	4	100	67 ± 2	68 ± 7	arterial	yes	e	[42]
Bovill and Sebel 1980	60.0		iv (2 min)	5	40	57 (45–65)	71 (53–87)	venous	yes	e	[41]
Christrup et al., 2008	1.5		iv (-)	7	43	24 (22–28)	68 (52–82)	venous	yes	i	[43]
Duthie et al., 1986 (1)	1.4	100.0	iv (24 h + bolus)	10	-	61 ± 8	69 ± 12	venous	yes	e	[44]
Duthie et al., 1986 (2)	1.5	100.0	iv (24 h + bolus)	13	-	49 ± 14	65 ± 14	venous	yes	e	[44]
Duthie et al., 1986 (3)	1.4	100.0	iv (24 h + bolus)	10	-	58 ± 11	69 ± 11	venous	yes	e	[44]
Duthie et al., 1986 (4)	7.2	100.0	iv (26 h + bolus)	12	-	55 ± 12	69 ± 9	venous	yes	i	[44]
Gourlay et al., 1989	1.0		iv (1 min)	6	-	-	70 (40–85)	venous ^c^	no	e	[45]
Gupta et al., 1995		50.0	iv (48 h)	6	0	-	-	venous ^d^	no	i	[46]
Holley and van Steennis 1988 (1)	1.3	25.0	iv (loading dose + 24 h)	10	0	54 ± 12	76 ± 12	arterial	yes	i	[47]
Holley and van Steennis 1988 (2)	2.5	50.0	iv (loading dose + 24 h)	10	0	44 ± 15	81 ± 16	arterial	yes	e	[47]
Holley and van Steennis 1988 (3)	5.0	100.0	iv (loading dose + 24 h)	10	0	56 ± 12	80 ± 17	arterial	yes	e	[47]
Holley and van Steennis 1988 (4)	6.5	125.0	iv (loading dose + 24 h)	9	0	54 ± 12	77 ± 5	arterial	yes	i	[47]
Lim et al., 2012	1.5		iv (5 min)	22	58	23 (19–32)	67 (51–101)	venous	no	e	[48]
MacLeod et al., 2012	0.3		iv (5 sec)	10	51	25 (18–55)	73 ± 13	arterial	no	i	[49]
McClain and Hug 1980	6.4		iv (1.5 min)	5	0	- (22–29)	75 (65–85)	arterial	no	i	[40]
Saari et al., 2008 ^e^	5.0		iv (2 min)	12	42	-	-	venous	no	e	[6]
Saari et al., 2008 (DDI) ^e,f^	5.0		iv (2 min)	12	42	-	-	venous	no	e	[6]
Singleton et al., 1987 (1)	20.7		iv (2 min)	7	-	33 (18–41)	-	arterial	yes	e	[50]
Stoeckel et al., 1982	7.6		iv (bolus)	3	33	22 (20–26)	66 (59–77)	venous	no	e	[51]
Streisand et al., 1991	15.0		iv (8 min)	10	0	27 (23–31)	76 (68–85)	arterial	no	e	[52]
Varvel et al., 1989	11.4		iv (5 min)	8	63	45 (33–57)	68 (52–100)	arterial/venous	yes	e	[53]
Ziesenitz et al., 2015 ^e^	5.0		iv (10 min)	16	25	33 (22–49)	73 (61–85)	venous	no	i	[5]

-: not available, e: external test dataset, i: internal training dataset, iv: intravenous. Age and weight are reported as the mean with standard deviation or range if available. ^a^ Dose of bolus injection and short infusion, respectively; ^b^ dose of long-term infusion; ^c^ venous blood samples from a central venous catheter; ^d^ sample information was not specified and venous blood samples were assumed; ^e^ norfentanyl concentrations measured; ^f^ with concomitant administration of voriconazole.

**Table 2:** Overview of clinical studies used for evaluation of the pediatric PBPK model predictions of fentanyl.

Clinical Study ID	Dose ^a^ [µg/kg]	Dose ^b^ [µg/kg/h]	Administration	*n*	Female [%]	Chronological Age	Gestational Age	Weight [kg]	Blood Sample	Surgery	Reference
Collins et al., 1985	30.0		iv (1 min)	9	22	-	32 (23–38)	1.1 (0.7–1.6)	arterial	yes	[54]
Gauntlett et al., 1988 (1)	52.5		iv (2 min)	1	-	1 day	38	2.8	arterial	yes	[36]
Gauntlett et al., 1988 (2)	56.5		iv (2 min)	1	-	3 days	40	2.5	arterial	yes	[36]
Gauntlett et al., 1988 (all) ^c^	54.1 ± 2.3		iv (2 min)	14	-	18.0 (1–71) days	38 (32–40)	2.7 (1.9–3.9)	arterial	yes	[36]
Koehntop et al., 1986 (1)	25.0		iv (1–3 min)	1	-	2 days	-	2.0	arterial	yes	[37]
Koehntop et al., 1986 (2)	50.0		iv (1–3 min)	1	-	2 days	-	3.5	arterial	yes	[37]
Koehntop et al., 1986 (all) ^c^	10.0–50.0		iv (1–3 min)	14	-	3.0 (0.5–14) days	-	2.9 (1.9–3.8)	arterial	yes	[37]
Saarenmaa et al., 2000 ^c^	10.5	1.5	iv (1 h + 58 h)	38	26	0.4 (0–2) days	32 (26–42)	1.8 (0.9–3.6) ^d^	arterial	yes	[38]
Singleton et al., 1987 (2)	31.2		iv (2 min)	7	-	6.5 (3–10) months	-	-	arterial	yes	[50]
Singleton et al., 1987 (3)	30.8		iv (2 min)	7	-	2.7 (1–9) years	-	-	venous ^e^	yes	[50]

-: not available, iv: intravenous. Age and weight are reported as the mean with range if available. ^a^ Dose of bolus injection and short infusion, respectively; ^b^ dose of long-term infusion; ^c^ studies from which observed individual clearance values were extracted; ^d^ median and range; ^e^ venous blood samples from a central venous catheter.

**Table 3:** Fentanyl and norfentanyl drug-dependent model parameters.

Parameter	Fentanyl					Norfentanyl					Description
	Value	Unit	Source	Literature	Reference	Value	Unit	Source	Literature	Reference	
MW	336.5	g/mol	lit.	336.5	[55] ^a^	232.3	g/mol	lit.	232.3	[55] ^b^	Molecular weight
pK_a_ (base)	8.99		lit.	8.99	[55] ^a^	10.03		lit.	10.03	[55] ^b^	Acid dissociation constant
logP	3.49		lit.	3.49	[56]	2.00		lit.	2.00	[55] ^b^	Lipophilicity
f_u_ (adults)	20.8	%	lit.	20.8	[57]	81.9	%	lit.	81.9	[58]	Unbound fraction
f_u_ (pediatrics)	29.0–33.0	%	calc.		[34]						Unbound fraction
CYP3A4 K_m_ → norfen	117	µmol/L	lit.	117	[27]						Michaelis–Menten constant
CYP3A4 k_cat_ → norfen	20.6	1/min	optim.	-	-						Catalytic rate constant
CYP3A7 K_m_ → norfen	596	µmol/L	calc.^c^	-	-						Michaelis–Menten constant
CYP3A7 k_cat_ → norfen	5.22	1/min	calc.^c^	-	-						Catalytic rate constant
Unspecific hepatic clearance → undef	1.46	1/min	lit.	-	-						Elimination from plasma (first-order process in the liver)
P-gp K_m_	5.72	µmol/L	optim.	-	-						Michaelis–Menten constant
P-gp k_cat_	1.71	1/min	optim.	-	-						Transport rate constant
B/P ratio	0.87		lit.	0.87	[59]	1.26		calc.	-	-	Blood-to-plasma ratio
GFR fraction	1.00		asm.	-	-	4.30		optim.	-	-	Filtered drug in the urine
Partition coefficients	Diverse ^d^		calc.	R&R	[60,61,62]	Diverse ^d^		calc.	Schmitt	[63]	Cell to plasma partitioning
Cellular permeability	Diverse ^d^	cm/min	calc.	Ch.-dep. Schmitt	[31]	1.80 × 10^−2^	cm/min	calc.	PK-Sim	[31]	Perm. into the cellular space
CYP3A4 K_I_ of voriconazole	9.33	µmol/L	lit.	9.33	[39]						The inhibitor concentration when reaching half of k_inact_
CYP3A4 k_inact_ of voriconazole	0.015	1/min	lit.	0.015	[39]						The maximum inactivation rate constant

-: not available; asm.: assumed; calc.: calculated; Ch.-dep. Schmitt: Charge-dependent Schmitt calculation method; CYP: cytochrome P450; GFR: glomerular filtration rate; lit.: literature; norfen: norfentanyl; optim.: optimized; P-gp: P-glycoprotein; Perm.: permeability; PK-Sim: PK-Sim standard calculation method; Schmitt: Schmitt calculation method; R&R: Rodgers and Rowland calculation method; undef: undefined metabolite; ^a^ DrugBank entry for fentanyl: https://www.drugbank.ca/drugs/DB00813, accessed 30 July 2020; ^b^ DrugBank entry for norfentanyl: https://www.drugbank.ca/metabolites/DBMET00341, accessed 30 July 2020; ^c^ for detailed information on calculations, please refer to Section 1 in the Supplementary Materials; ^d^ different values for different organs estimated by the corresponding calculation method.

## Supplementary Materials

The following are available online at https://www.mdpi.com/1999-4923/12/10/908/s1, Electronic Supplementary Materials: Additional model information including detailed model evaluation.
