# Supplementary material for: Physiologically-Based Pharmacokinetic (PBPK) Modeling Providing Insights into Fentanyl Pharmacokinetics in Adults and Pediatric Patients
Source: Pharmaceutics. 2020 Sep 23;12(10):908. doi: 10.3390/pharmaceutics12100908 (PMC7598194; doi:10.3390/pharmaceutics12100908)
Supplement: Supplementary file 1 [file pharmaceutics-12-00908-s001.pdf]

Pharmaceutics

Supplementary Materials: PBPK Modeling Providing Insights into Fentanyl Pharmacokinetics in Adults and Pediatric Patients

Lukas Kovar, Andreas Weber, Michael Zemlin, Yvonne Kohl, Robert Bals, Bernd Meibohm, Dominik Selzer and Thorsten Lehr

Contents

1 PBPK Model Building **2**

1.1 PBPK Model Building – General . . . 2

1.2 Clearance in Neonates with Increased Intraabdominal Pressure . . . 3

1.3 System-dependent Parameters and Virtual Populations . . . 4

2 Drug-Drug-Interaction (DDI) Modeling **6**

2.1 DDI Modeling – General . . . 6

2.2 Mathematical Implementation of DDIs . . . 6

2.2.1 Competitive Inhibition . . . 6

2.2.2 Mechanism-Based Inhibition (MBI) . . . 6

3 PBPK Model Evaluation **8**

3.1 Adult PBPK Model Evaluation . . . 9

3.2 Pediatric PBPK Model Evaluation . . . 17

3.3 Quantitative PBPK Model Evaluation . . . 21

3.4 Mean Relative Deviation (MRD) Values of Fentanyl and Norfentanyl Plasma Concentration Predictions . . . 21

3.5 Geometric Mean Fold Error (GMFE) of AUC_{last} Predictions . . . 23

3.6 Fentanyl and Norfentanyl PBPK Model Sensitivity Analysis . . . 24

References **26**

1 PBPK Model Building

1.1 PBPK Model Building – General

For *a priori* physiologically-based pharmacokinetic (PBPK) predictions in pediatrics, a common workflow is to first model and evaluate the PBPK model with published pharmacokinetics (PK) data in adults. Subsequently, the model is extrapolated to pediatric populations [1–5]. While the general model building process is depicted in the methods section of the main manuscript, this section provides additional model information.

The fentanyl PBPK model includes the metabolic pathway of fentanyl to the inactive metabolite norfentanyl via Cytochrome P450 (CYP) 3A4 and CYP3A7 [6], an unspecific hepatic clearance metabolizing fentanyl to other non-specified metabolites, distribution and excretion via P-glycoprotein (P-gp), as well as renal excretion through glomerular filtration [7–9]. The involvement of CYP3A7 in the metabolic elimination of fentanyl is still unclear [6, 10]. As CYP3A4 and CYP3A7 share a similar substrate spectrum [5, 11] and since CYP3A7 is the major fetal form of CYP3A [11], this CYP enzyme might be important for PK predictions of fentanyl in pediatric populations. Hence, CYP3A7 was incorporated in the model. Unfortunately, K_m and V_{max} values for the metabolism of fentanyl via CYP3A7 have not been published in the literature. Yet, a study by Williams and colleagues provided information on the relative metabolic capabilities of CYP3A4 and CYP3A7 to metabolize various molecules (n=15) and compared respective K_m [µmol/L] and V_{max} [nmol/min/nmol P450] values [11]. The dataset was further expanded with the K_m and V_{max} values from three more molecules [12]. On average, K_m values for CYP3A7 were 5.1 times higher in comparison to the corresponding K_m values of CYP3A4 for the investigated substances, while V_{max} values appeared to be 75% lower. Subsequently, these factors were utilized and multiplied with the K_m and k_{cat} values for the metabolism of fentanyl through CYP3A4 (117 µmol/L and 20.6 1/min) in order to obtain the model input parameters for CYP3A7. This resulted in a K_m value of 596 µmol/L and a k_{cat} value of 5.22 1/min.

In addition, various *in vitro* and animal studies as well as a DDI study with quinidine suggest that fentanyl is a substrate of P-gp [13–16]. As a consequence, fentanyl was implemented to be a substrate of the transport protein P-gp in the developed PBPK model. In contrast, there were no information, which state that norfentanyl is a substrate of P-gp. As a result, norfentanyl was not implemented as a substrate of the transport protein P-gp. Since norfentanyl is predominantly eliminated via urine, a renal clearance was implemented [8]. Parameter optimization yielded a glomerular filtration rate fraction of 4.3 indicating tubular secretion in the PBPK model.

Table S1: Tissue-plasma partition coefficients of the final fentanyl PBPK model.

	Fentanyl[a]				Norfentanyl[b]
Organ	Adults	Neonates	Infants	Children	Adults
Bone	1.43	1.46	1.39	1.27	2.11
Brain	1.55	1.97	1.90	1.73	28.53
Fat	2.15	1.82	2.05	2.09	1.37
Gonads	4.07	3.51	3.31	3.04	11.77
Heart	3.68	3.13	2.95	2.72	21.02
Kidney	7.77	6.26	5.88	5.43	16.76
Large Intestine	4.40	4.09	3.87	3.55	8.15
Liver Pericentral	7.13	5.83	5.47	5.05	19.23
Liver Periportal	7.13	5.83	5.47	5.05	19.23
Lung	6.19	5.11	4.81	4.43	9.27
Muscle	4.21	3.77	3.58	3.28	2.81
Pancreas	3.38	3.34	3.18	2.91	4.31
Saliva	0.21	0.33	0.32	0.29	0.82
Skin	3.10	3.33	3.19	2.90	6.70
Small Intestine	4.40	4.09	3.87	3.55	8.15
Spleen	4.94	4.01	3.76	3.47	6.86
Stomach	4.40	4.09	3.87	3.55	8.15

Partition coefficients between intracellular space and plasma.
Mean ages of the adult, child, infant, and neonate population were 32 years, 2.7 years, 6.5 months, and 0.4 days, respectively, adapted from [8, 17, 18]
[a] Estimated via Rodgers and Rowland [19–21]
[b] Estimated via Schmitt [22]

1.2 Clearance in Neonates with Increased Intraabdominal Pressure

In the gathered pediatric clinical trial data, several of the neonates showed a significantly reduced fentanyl clearance [18, 23, 24]. It was hypothesized that this might partly be due to an increased intra-abdominal pressure resulting in a decreased hepatic clearance [18, 23, 24]. The plasma concentration-time profiles of four of these patients were depicted in the study by Gauntlett et al. and Koehntop et al., respectively [23, 24]. The profiles were digitized and used as an internal training dataset. In order to account for the reduced elimination, a factor was estimated for each patient and multiplied with the catalytic rate constant values for CYP3A4 and CYP3A7 as well as with the unspecific hepatic clearance. The resulting factors are shown in Table S2. The arithmetic mean of these factors was then used to adapt the clearance of the remaining 6 patients with proposed increased intraabdominal pressure. If no information on intraabdominal pressure was available in a study, the clearance was adapted for all patients with abdominal surgery [23].

Table S2: Estimated factors for clearance adjustment in pediatric patients who had abdominal surgery.

Study ID	Estimated Factor	Study Reference
Gauntlett et al. (1)	0.168	[23]
Gauntlett et al. (2)	0.148	[23]
Koentrop et al. (1)	0.089	[24]
Koentrop et al. (2)	0.259	[24]
**Arithmetic mean**	**0.166**	

1.3 System-dependent Parameters and Virtual Populations

The demographic characteristics of the study populations (see Tables 1–2 in the main manuscript) were used to create virtual individuals with the respective system-dependent physiological parameters such as blood flow rates and organ compositions in PK-Sim®. The applied algorithms for the generation of these virtual individuals have been previously reported [25]. If no information on the patient demographics were available, a 30-year-old male was assumed with body weight, height and BMI values according to the PK-Sim® database.

As Stader and colleagues pointed out, for most anatomical, physiological, and biological parameters, a sample size of at least 100 individuals is recommended [26]. For system parameters with high variability, such as enzyme and transporter abundance, a virtual population containing 500 individuals might be more appropriate [26]. Simulations with n=100 and n=500 for various dosing regimens (i.e. including iv bolus, short infusions and long-term infusions) were tested resulting in negligibly small differences in simulated plasma concentration-time profiles. Thus, predictions with virtual populations were simulated with 100 individuals.

Virtual individuals were generated for virtual populations according to the respective population demographics (see Tables 1–2 in the main manuscript) for each study separately. Demographics of virtual individuals (i.e. age, height, weight and corresponding organ volumes, tissue compositions, blood flow rates, etc.) were varied by an implemented algorithm in PK-Sim® within the limits of the ICRP (International Commission on Radiological Protection) and NHANES (National Health and Nutrition Examination Survey) databases, respectively [27, 28]. If no age range was reported in the clinical trial with adult patients, virtual populations were created with individuals 20 to 50 years of age and without specific weight or height restrictions as implemented in PK-Sim®. Tissue expression distributions of enzymes and proteins were used according to the PK-Sim® expression database [29–31].

Furthermore, expression variability of the implemented enzymes (i.e. CYP3A4 and CYP3A7) and of the transport protein P-gp was implemented. System-dependent parameters, such as information on reference concentrations and the respective variabilities of enzymes and transporters are shown in Table S3.

Table S3: System-dependent parameters and expression of relevant enzymes and transporters.

Enzyme / Transporter	Mean reference concentration [µmol/L][a]	GSD of the reference concentration in adults[b]	Relative expression in different organs[c]	Ontogeny function	Half-life liver [hours]	Half-life Intestine [hours]
**Enzymes**						
CYP3A4	4.32 [32]	1.18 (liver)[33]<br>1.45 (intestine)[33]	RT-PCR [29]	[33]	36	23
CYP3A7	7.98 [34]	1.25 [33]	RT-PCR [29]	[33]	36	23
**Transporters**						
P-gp	1.00[d]	1.70, 1.84, 1.78, 1.60 [35][e]	RT-PCR [30][f]	[36][g]	36	23
**Processes**						
Unspecific hepatic clearance of fentanyl	-	1.40[h]		-		

CYP: cytochrome P450, **GSD**: Geometric standard deviation, **P-gp**: P-glycoprotein, **RT-PCR**: reverse transcription polymerase chain reaction

[a] [µmol protein/L] in the tissue of the highest expression

[b] for information on geometric standard deviation in pediatrics, please refer to [33]

[c] PK-Sim® expression database profile

[d] reference concentration was set to 1.0 µmol/L and k_{cat} optimized according to [37]

[e] geometric standard deviations for neonates, infants, children and adults, respectively, according to [36]

[f] with the relative expression in intestinal mucosa increased by factor 3.57 according to [38]

[g] since no specific ontogeny function for P-gp is implemented in PK-Sim®, the ontogeny function from Prasad et al. was used [36]

[h] geometric standard deviation with coefficient of variation (CV) of 35 % assumed

2 Drug-Drug-Interaction (DDI) Modeling

2.1 DDI Modeling – General

Voriconazole is an inhibitor of two CYP enzymes, CYP3A4 and CYP2C9. While voriconazole inhibits CYP2C9 competitively, it acts as both a competitive and mechnism-based inhibitor in case of CYP3A4 [39]. For the assessment of the DDI with voriconazole a previously developed voriconazole PBPK model was used [39]. Voriconazole shows dose- and time-dependent nonlinear pharmacokinetics which was well captured in the simulations of the used voriconazole PBPK model [39]. The parameters of the model can be found in the respective publication [39].
The DDI simulations presented in the manuscript are pure predictions. The DDI study was not used for model input parameter estimation during fentanyl and norfentanyl PBPK model development. Interaction parameters necessary for DDI simulation were obtained from the published DDI perpetrator PBPK model. With that, the adult PBPK model could not only be evaluated by its predictive performance with the test dataset but also by prediction of a DDI study [7].

2.2 Mathematical Implementation of DDIs

2.2.1 Competitive Inhibition

Competitive inhibition describes the competition of substrate and inhibitor for reversibly binding to the active site of an enzyme or transporter. The inhibition can be overcome by high substrate concentrations leading to a concentration-dependency of the inhibition. Hence, the maximum reaction velocity $\mathrm{V_{max}}$ is not affected during a competitive inhibition, while $\mathrm{K_m}$ is increased through the inhibition process yielding $\mathrm{K_{m,app}}$ (Equation S1). The reaction velocity (v) for the substrate during concomitant administration with a competitive inhibitor is described by Equation S2 [33]:

$$K_{m,app} = K_m \cdot \left(1 + \frac{[I]}{K_i}\right) \tag{S1}$$

$$v = \frac{V_{max} \cdot [S]}{K_{m,app} + [S]} \tag{S2}$$

with $K_{m,app}$ = Michaelis-Menten constant in the presence of inhibitor, K_m = Michaelis-Menten constant, $[I]$ = free inhibitor concentration, K_i = dissociation constant of the inhibitor-enzyme/transporter complex, v = reaction velocity, V_{max} = maximum reaction velocity, $[S]$ = free substrate concentration.

2.2.2 Mechanism-Based Inhibition (MBI)

While competitive inhibition is a reversible mechanism, mechanism-based inhibition (MBI) is an irreversible type of inhibition. *De novo* synthesis of the inactivated protein and clearance of the mechanism-based inactivator is required for the enzyme or transporter to return to baseline activity (time-dependency). During an MBI the protein degradation rate constant ($\mathrm{k_{deg}}$) is increased yielding $\mathrm{k_{deg,app}}$ (Equation S3), while the synthesis ($\mathrm{R_{syn}}$) is not affected by the inhibition process. The protein turnover during MBI is described by Equation S4. In addition, as mechanism-based inactivators are also competitive inhibitors, the $\mathrm{K_m}$ in the Michaelis-Menten reaction velocity equation is substituted by $\mathrm{K_{m,app}}$ as in Equation S5 [33]:

$$k_{deg,app} = k_{deg} + \left(\frac{k_{inact} \cdot [I]}{K_I + [I]} \right) \tag{S3}$$

$$\frac{dE(t)}{dt} = R_{syn} - k_{deg,app} \cdot E(t) \tag{S4}$$

$$v = \frac{V_{max} \cdot [S]}{K_{m,app} + [S]} = \frac{k_{cat} \cdot E(t) \cdot [S]}{K_{m,app} + [S]} \tag{S5}$$

with $k_{deg,app}$ = enzyme or transporter degradation rate constant in the presence of mechanism-based inactivator, k_{deg} = enzyme or transporter degradation rate constant, k_{inact} = maximum inactivation rate constant, $[I]$ = free inactivator concentration, K_I = concentration for half-maximal inactivation, $E(t)$ = enzyme or transporter concentration, R_{syn} = rate of enzyme or transporter synthesis, v = reaction velocity, V_{max} = maximum reaction velocity, $[S]$ = free substrate concentration, $K_{m,app}$ = Michaelis-Menten constant in the presence of inactivator, k_{cat} = catalytic rate constant.

Hereby, k_{deg} can be computed from the half-lives ($t_{1/2}$) of the specific enzyme, which are depicted in Table S2, with $k_{deg} = \ln(2)/t_{1/2}$. Moreover, R_{syn} is calculated by $R_{syn} = E_{0,Enzyme} \cdot k_{deg}$, with $E_{0,Enzyme}$ being the amount of this enzyme in the tissue of interest before mechanism-based inhibition.

3 PBPK Model Evaluation

The descriptive and predictive performance of the developed adult and pediatric PBPK models is comprehensively depicted in this section. Semilogarithmic and linear plots of plasma concentration-time profiles (population predictions) are compared to the profiles observed for both adult and pediatric PBPK models in Figures S1, S2, S5 and S6. Additionally, plots of population predictions of fractions of fentanyl excreted unchanged in urine (linear plots) are compared to measured values in Figure S2. Moreover, goodness-of-fit plots comparing predicted to observed plasma concentrations are shown in Figures S3 and S7.

Predicted compared to observed area under the plasma concentration-time curves from the first to the last data point (AUC_{last}) values are depicted in Figures S4 and S8.

The mean relative deviation (MRD) values as well as the predicted and observed AUC_{last} values including the geometric mean fold errors (GMFE) are listed in Tables S4 and S5. Local sensitivity analyses were performed with the PBPK model for adult, child, infant, full-term neonate and preterm neonate subpopulations. Detailed descriptions and the results of the sensitivity analyses are shown in Section 3.6.

3.1 Adult PBPK Model Evaluation

In this section, semilogarithmic and linear plots of plasma concentration-time profiles, linear plots of fractions of fentanyl dose excreted unchanged in urine (Figures S1 and S2), a goodness-of-fit plot of predicted compared to observed plasma concentrations (Figure S3) and a goodness-of-fit plot of predicted compared to observed AUC_{last} values (Figure S4) after intravenous administration of fentanyl in adults are shown.

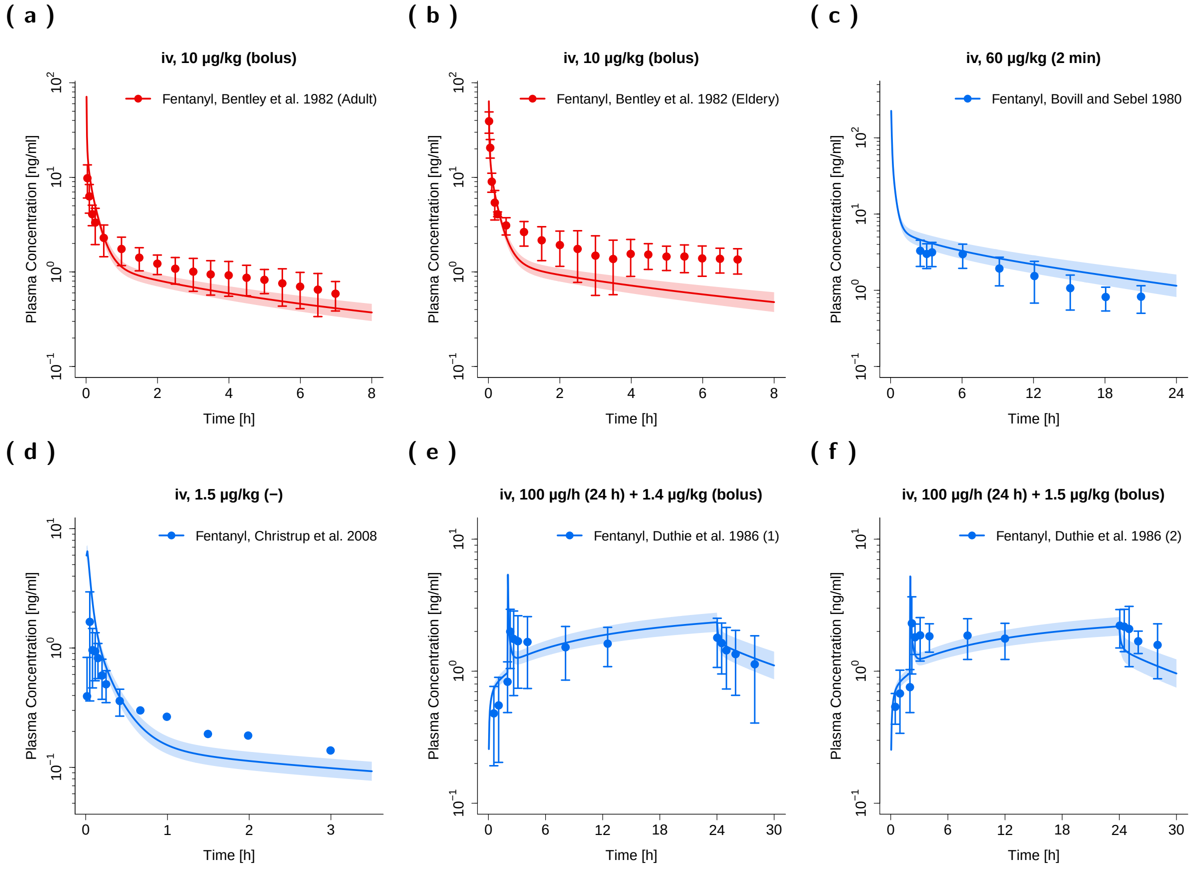

Figure S1: Fentanyl (blue: venous blood, darkblue: venous blood from central venous catheters, red: arterial blood) and norfentanyl (green: venous blood) plasma concentration-time profiles (semilogarithmic) after intravenous administration of fentanyl in adults. Observed data are shown as circles, if available ± standard deviation (SD). Population simulation (n=100) geometric means are shown as lines; the shaded areas represent the predicted population geometric SD. References with numbers in parentheses link to a specific observed dataset ID described in the study table (Table 1 in the main manuscript). Predicted and observed AUC_{last} values are compared in Table S5. **DDI**, drug-drug-interaction; **iv**, intravenous.

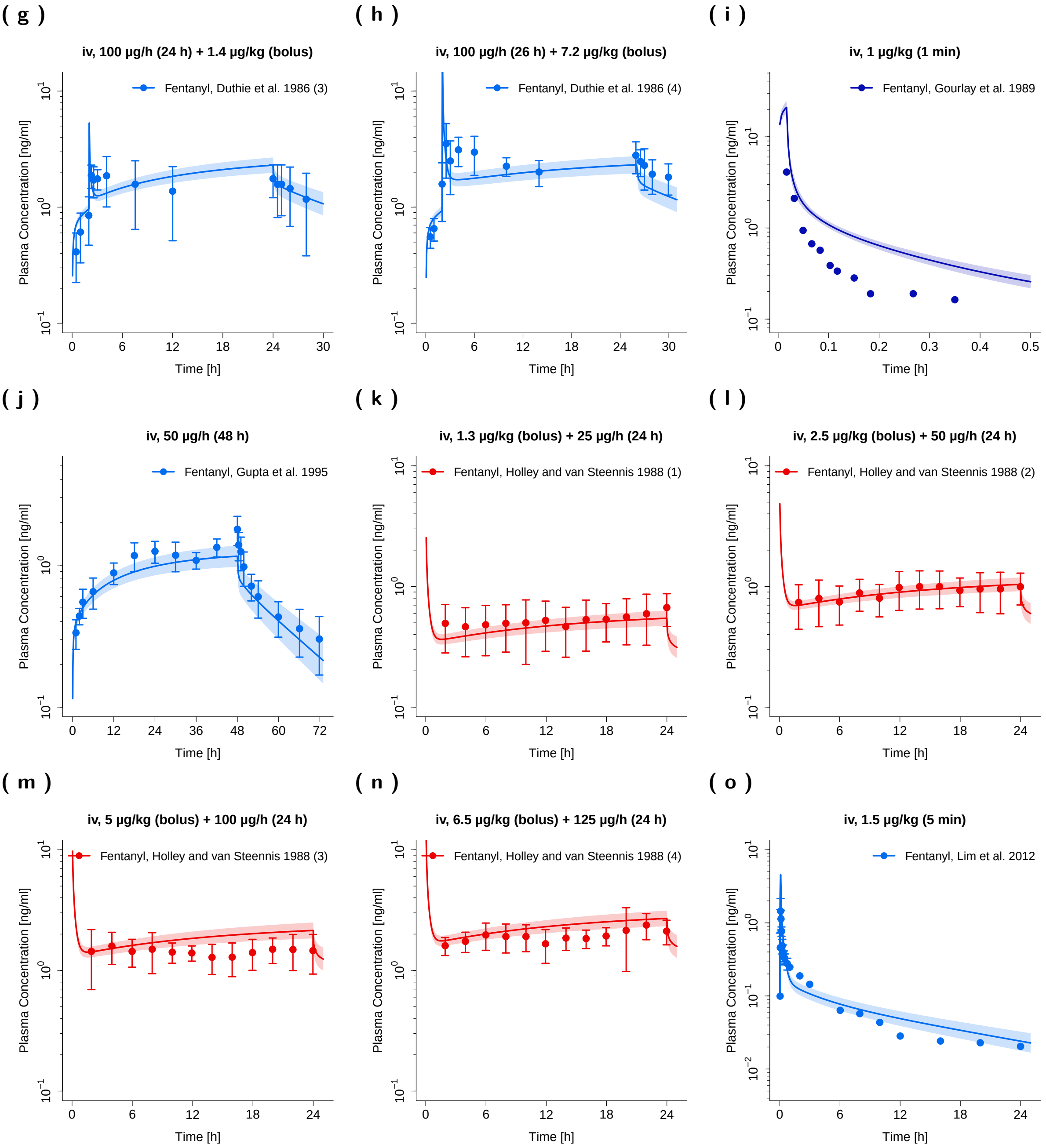

Figure S1: Fentanyl (blue: venous blood, darkblue: venous blood from central venous catheters, red: arterial blood) and norfentanyl (green: venous blood) plasma concentration-time profiles (semilogarithmic) after intravenous administration of fentanyl in adults. Observed data are shown as circles, if available $\pm$ standard deviation (SD). Population simulation (n=100) geometric means are shown as lines; the shaded areas represent the predicted population geometric SD. References with numbers in parentheses link to a specific observed dataset ID described in the study table (Table 1 in the main manuscript). Predicted and observed AUC_{last} values are compared in Table S5. **DDI**, drug-drug-interaction; **iv**, intravenous.(continued)

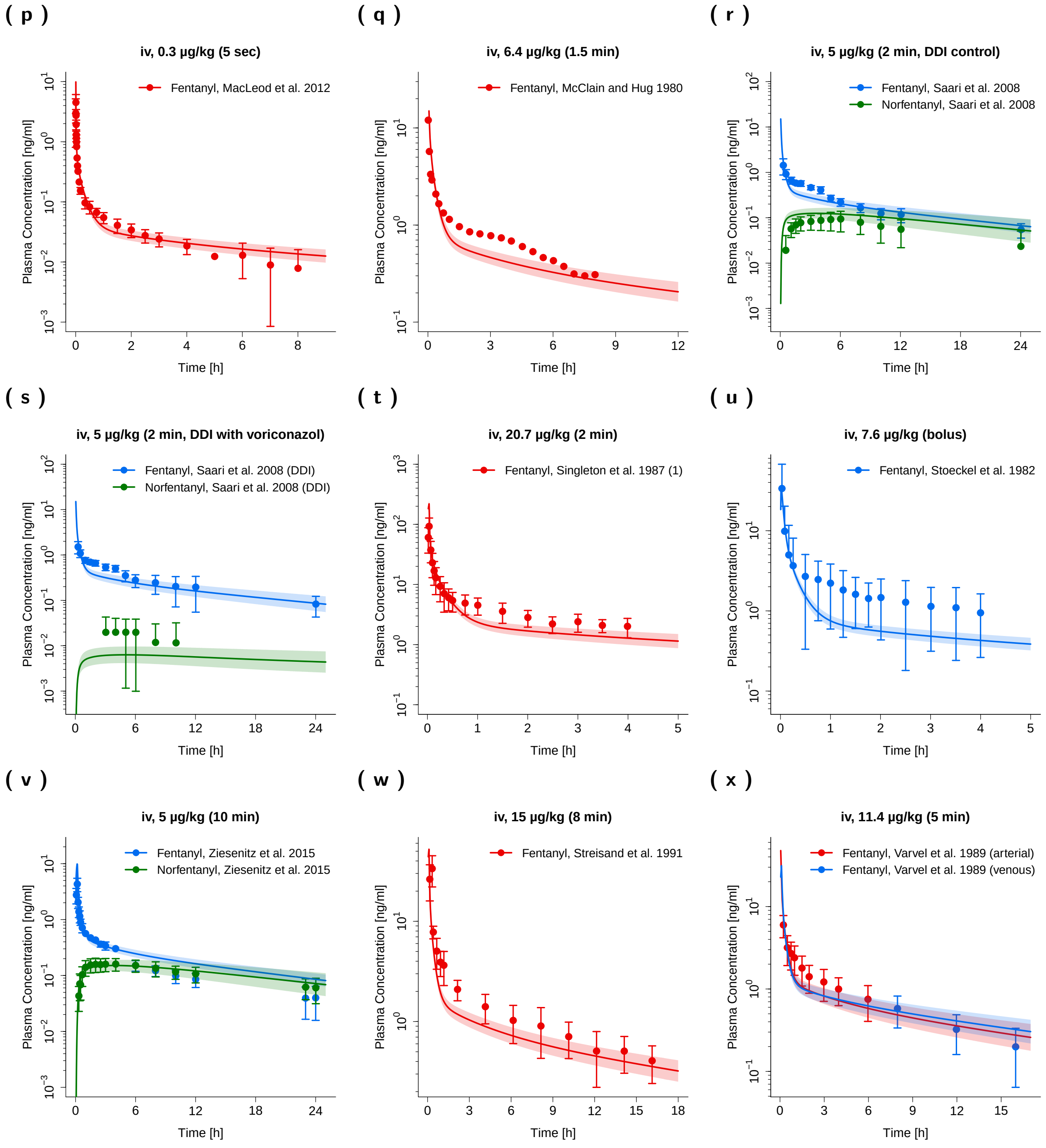

Figure S1: Fentanyl (blue: venous blood, darkblue: venous blood from central venous catheters, red: arterial blood) and norfentanyl (green: venous blood) plasma concentration-time profiles (semilogarithmic) after intravenous administration of fentanyl in adults. Observed data are shown as circles, if available ± standard deviation (SD). Population simulation (n=100) geometric means are shown as lines; the shaded areas represent the predicted population geometric SD. References with numbers in parentheses link to a specific observed dataset ID described in the study table (Table 1 in the main manuscript). Predicted and observed AUC_{last} values are compared in Table S5. **DDI**, drug-drug-interaction; **iv**, intravenous.(continued)

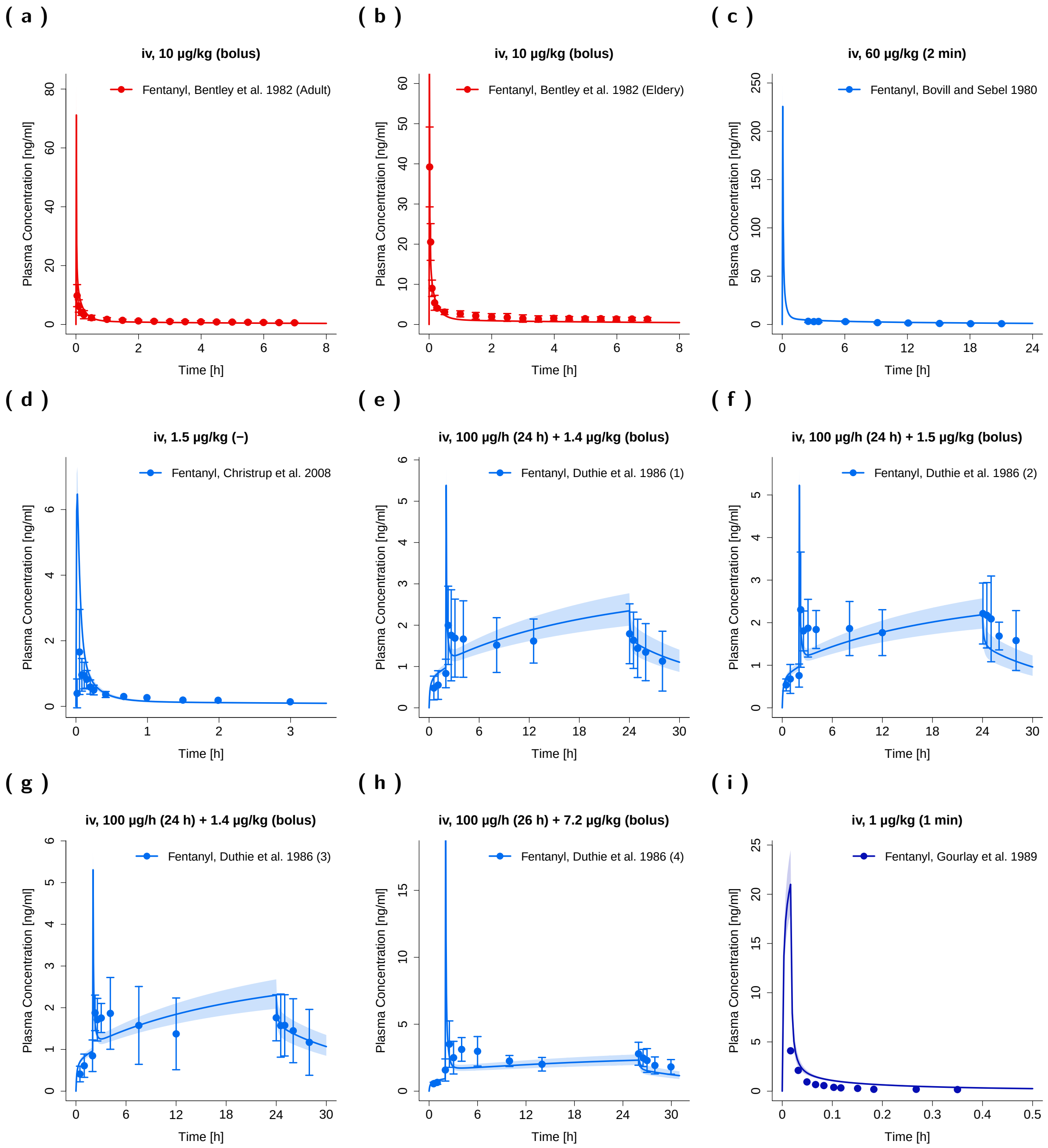

Figure S2: Fentanyl (blue: venous blood, darkblue: venous blood from central venous catheter, red: arterial blood) and norfentanyl (green: venous blood) plasma concentration-time profiles (linear) as well as fraction of fentanyl dose excreted unchanged in urine (yellow) after intravenous administration of fentanyl in adults. Observed data are shown as circles, if available ± standard deviation (SD). Population simulation (n=100) geometric means are shown as lines; the shaded areas represent the predicted population geometric SD. References with numbers in parentheses link to a specific observed dataset ID described in the study table (Table 1 in the main manuscript). Predicted and observed AUC_{last} values are compared in Table S5. **DDI**, drug-drug-interaction; **iv**, intravenous.

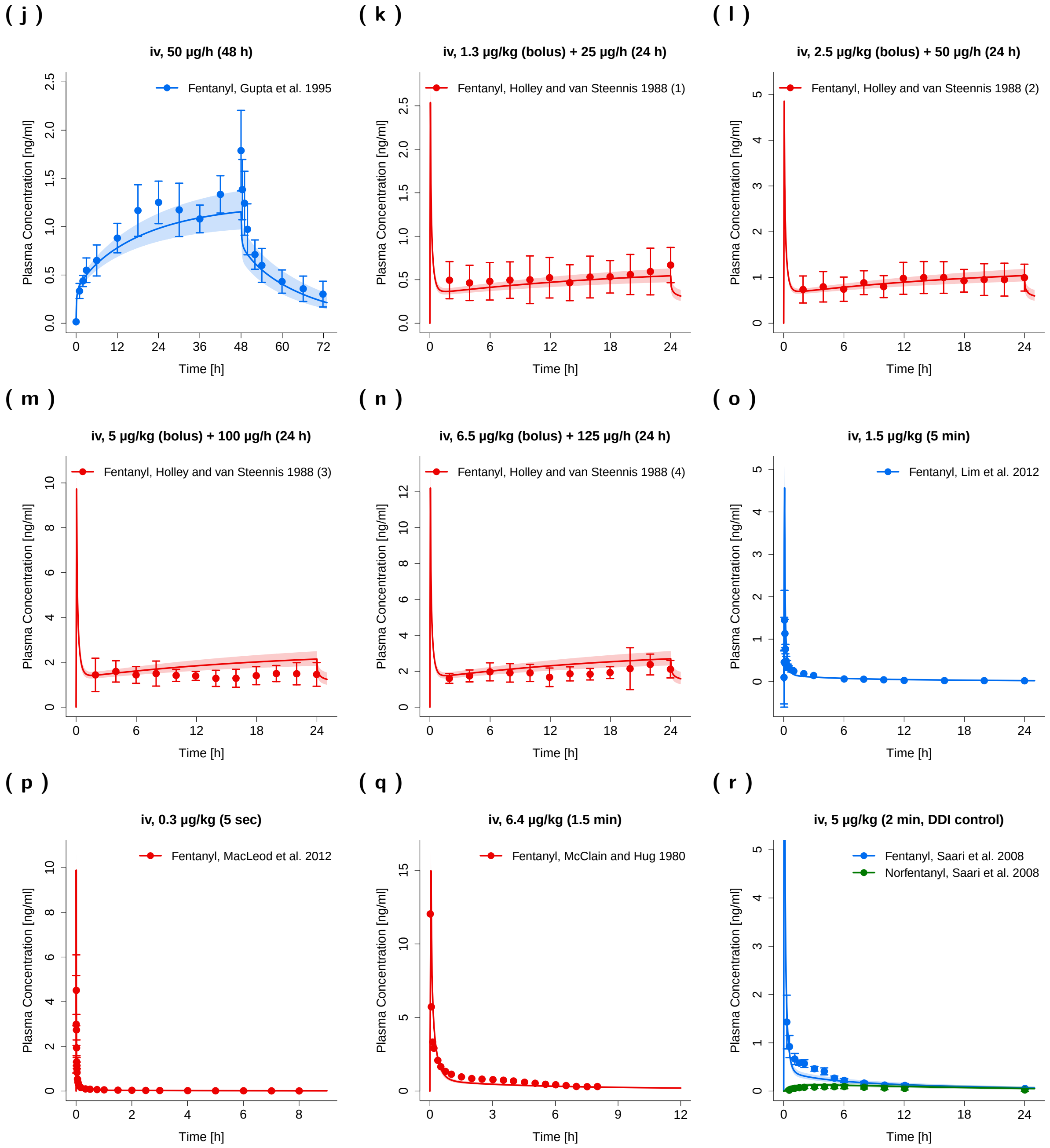

Figure S2: Fentanyl (blue: venous blood, darkblue: venous blood from central venous catheter, red: arterial blood) and norfentanyl (green: venous blood) plasma concentration-time profiles (linear) as well as fraction of fentanyl dose excreted unchanged in urine (yellow) after intravenous administration of fentanyl in adults. Observed data are shown as circles, if available ± standard deviation (SD). Population simulation (n=100) geometric means are shown as lines; the shaded areas represent the predicted population geometric SD. References with numbers in parentheses link to a specific observed dataset ID described in the study table (Table 1 in the main manuscript). Predicted and observed AUC_{last} values are compared in Table S5. **DDI**, drug-drug-interaction; **iv**, intravenous.(continued)

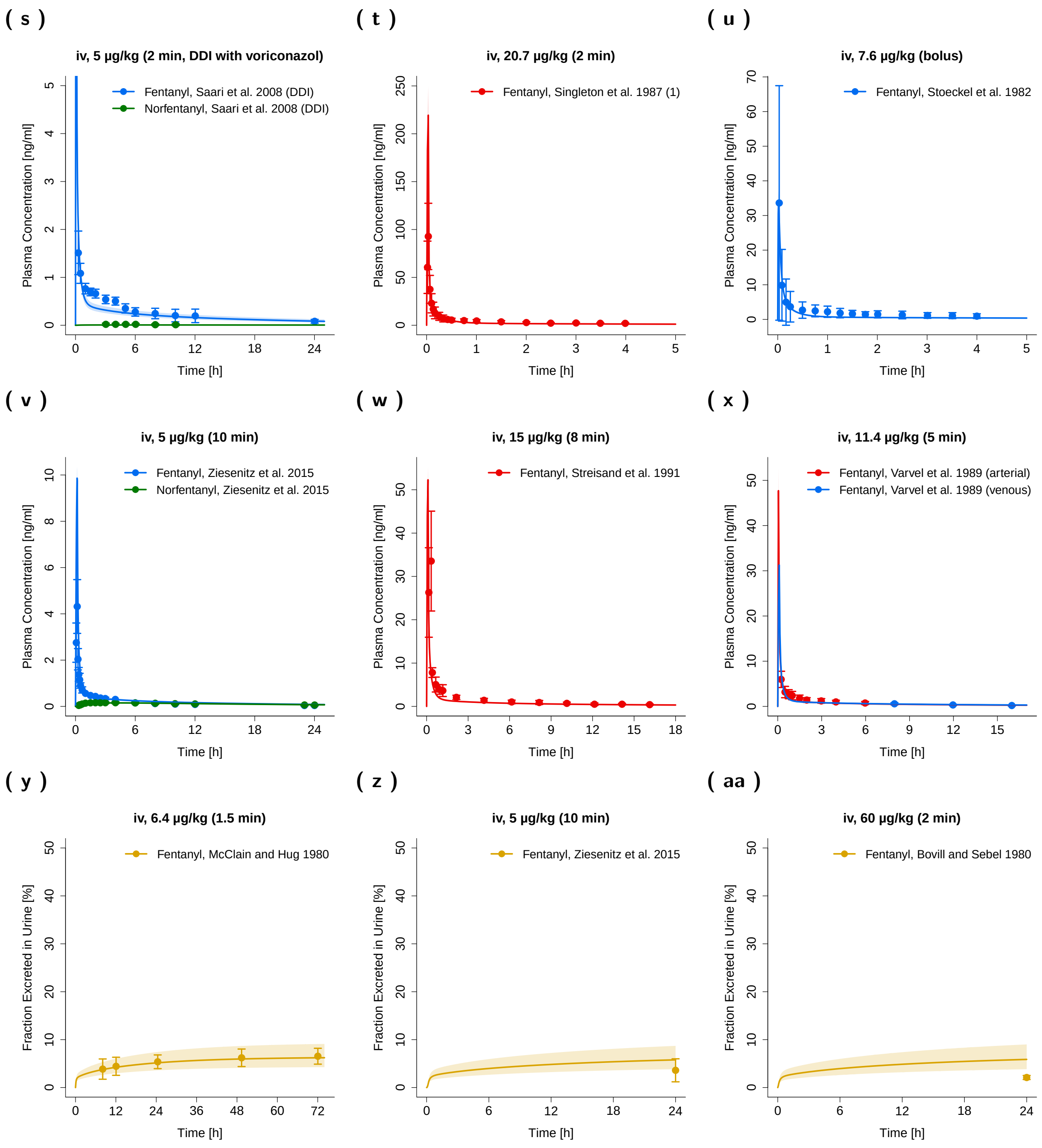

Figure S2: Fentanyl (blue: venous blood, darkblue: venous blood from central venous catheter, red: arterial blood) and norfentanyl (green: venous blood) plasma concentration-time profiles (linear) as well as fraction of fentanyl dose excreted unchanged in urine (yellow) after intravenous administration of fentanyl in adults. Observed data are shown as circles, if available ± standard deviation (SD). Population simulation (n=100) geometric means are shown as lines; the shaded areas represent the predicted population geometric SD. References with numbers in parentheses link to a specific observed dataset ID described in the study table (Table 1 in the main manuscript). Predicted and observed AUC_{last} values are compared in Table S5. **DDI**, drug-drug-interaction; **iv**, intravenous.(continued)

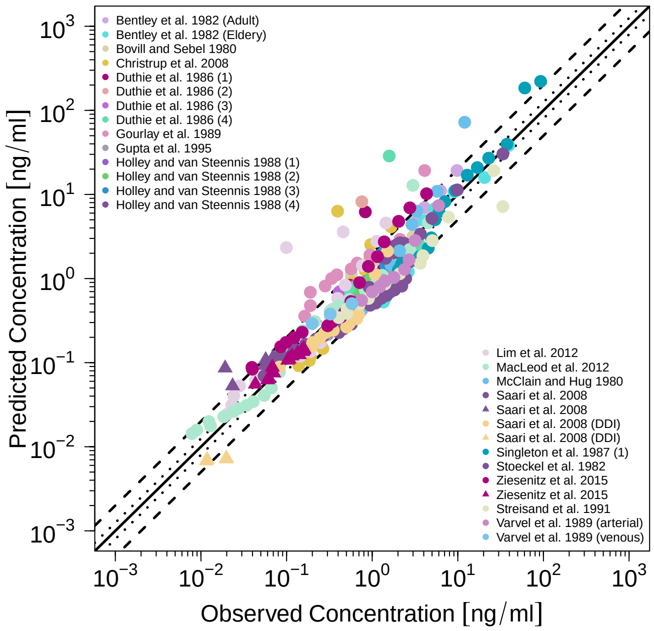

Figure S3: Predicted versus observed plasma concentrations of fentanyl and norfentanyl after intravenous administration of fentanyl in adults. Each symbol represents a single plasma concentration (circles: fentanyl, triangles: norfentanyl). The black solid line marks the line of identity. Black dotted lines indicate 1.25-fold, black dashed lines indicate 2-fold deviation.

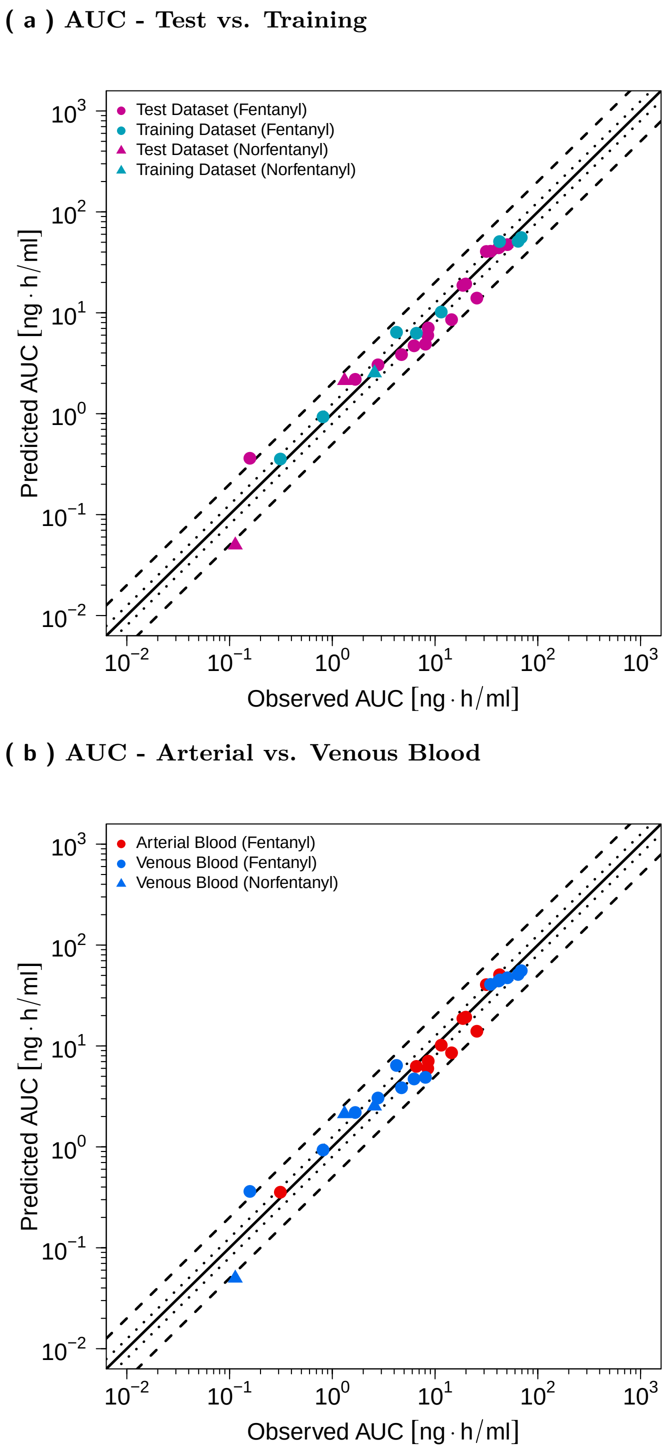

Figure S4: Predicted versus observed fentanyl and norfentanyl AUC values after intravenous administration of fentanyl in adults grouped by test and training dataset (a) and by arterial and venous blood samples (b). Each symbol represents the AUC_{last} of a different plasma profile. The black solid lines mark the lines of identity. Black dotted lines indicate 1.25-fold, black dashed lines indicate 2-fold deviation. **AUC**, area under the plasma concentration-time curve from the first to the last data point.

3.2 Pediatric PBPK Model Evaluation

In this section, semilogarithmic and linear plots of plasma concentration-time profiles (Figures S5 and S6), a goodness-of fit plot of predicted compared to observed plasma concentrations (Figure S7) and a goodness-of-fit plot of predicted compared to observed AUC_{last} values (Figure S8) after intravenous administration of fentanyl in pediatrics are shown.

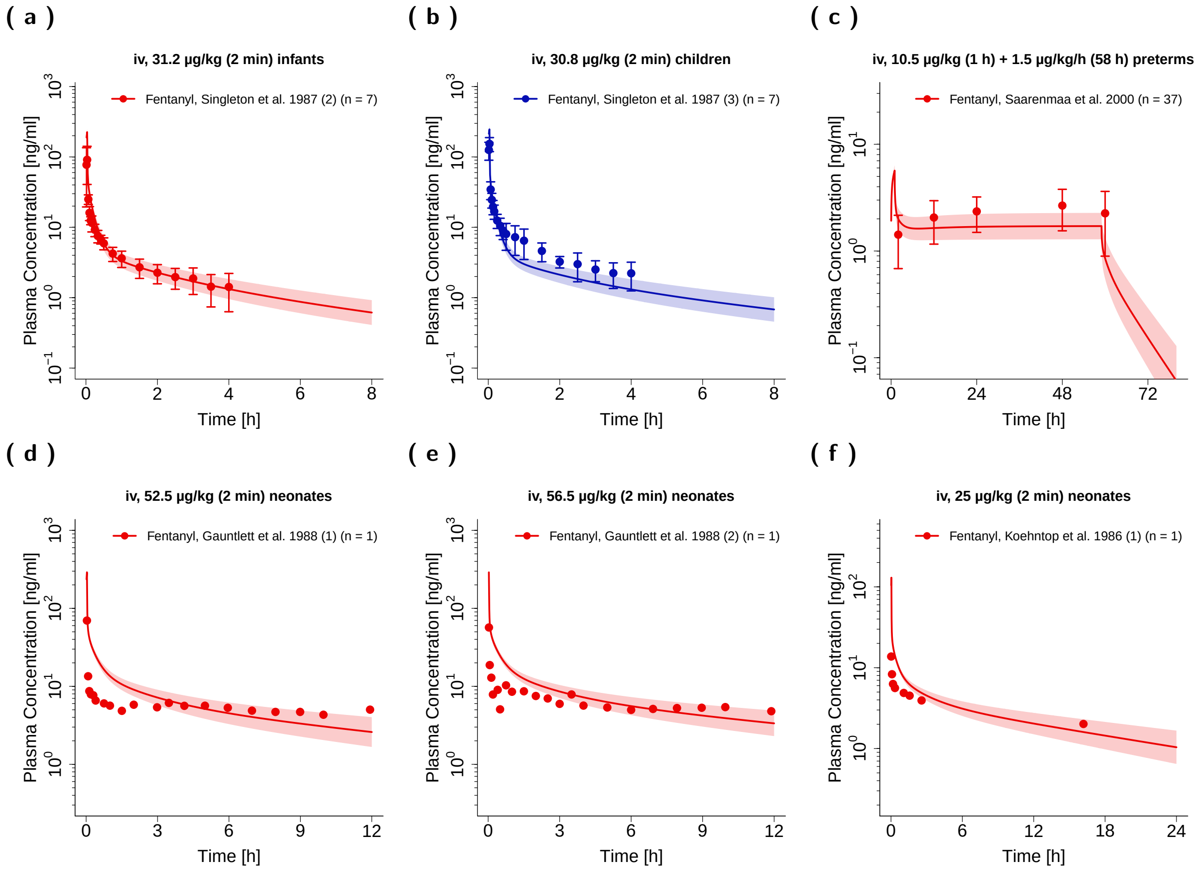

Figure S5: Fentanyl (darkblue: venous blood from central venous catheter, red: arterial blood) plasma concentration-time profiles (semilogarithmic) after intravenous administration of fentanyl in pediatrics. Observed data are shown as circles, if available ± standard deviation (SD). Population simulation (n=100) geometric means are shown as lines; the shaded areas represent the predicted population geometric SD. References with numbers in parentheses link to a specific observed dataset ID described in the study table (Table 2 in the main manuscript). Predicted and observed AUC_{last} are compared in Table S5. **iv**, intravenous.

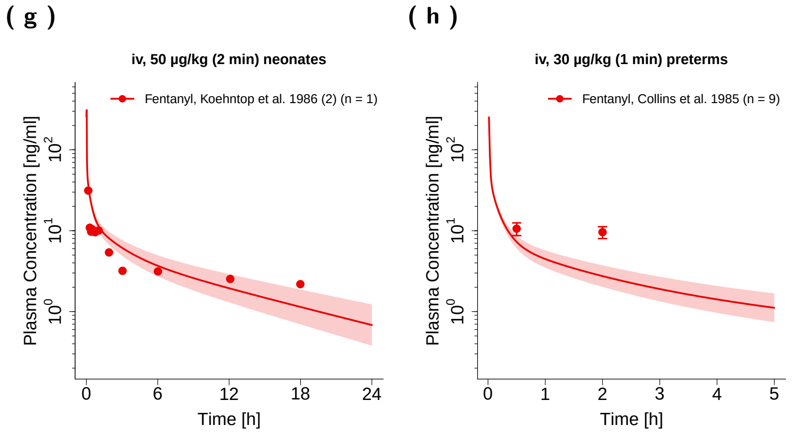

Figure S5: Fentanyl (darkblue: venous blood from central venous catheter, red: arterial blood) plasma concentration-time profiles (semilogarithmic) after intravenous administration of fentanyl in pediatrics. Observed data are shown as circles, if available ± standard deviation (SD). Population simulation (n=100) geometric means are shown as lines; the shaded areas represent the predicted population geometric SD. References with numbers in parentheses link to a specific observed dataset ID described in the study table (Table 2 in the main manuscript). Predicted and observed AUC_{last} are compared in Table S5. **iv**, intravenous.(continued)

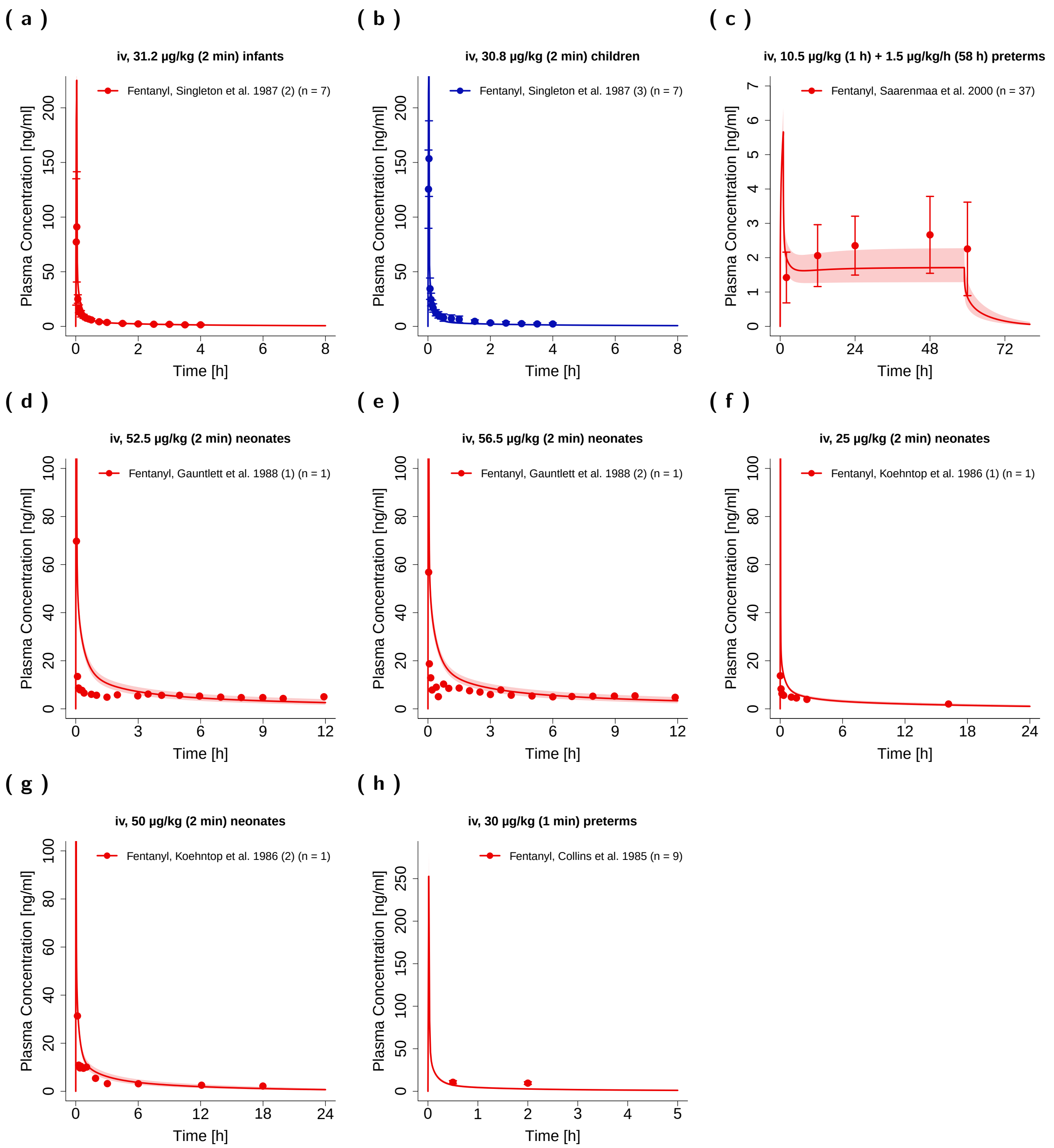

Figure S6: Fentanyl (darkblue: venous blood from central venous catheter, red: arterial blood) plasma concentration-time profiles (linear) after intravenous administration of fentanyl in pediatrics. Observed data are shown as circles, if available ± standard deviation (SD). Population simulation (n=100) geometric means are shown as lines; the shaded areas represent the predicted population geometric SD. References with numbers in parentheses link to a specific observed dataset ID described in the study table (Table 2 in the main manuscript). Predicted and observed AUC_{last} values are compared in Table S5. **iv**, intravenous.

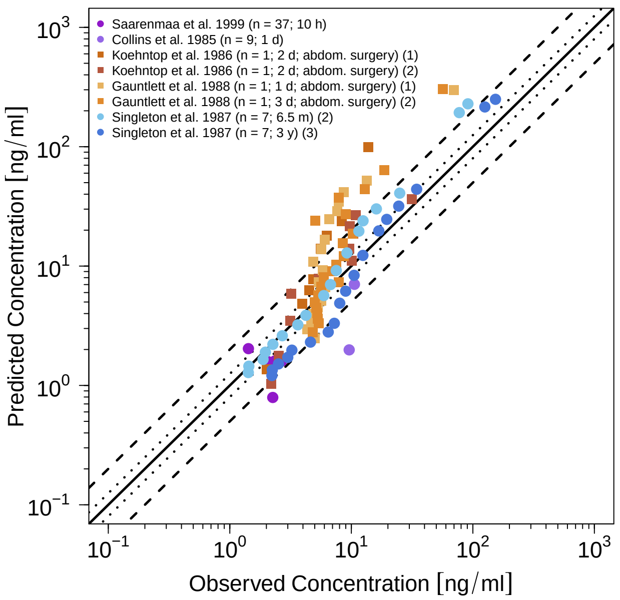

Figure S7: Predicted versus observed plasma concentrations of fentanyl for the pediatric PBPK model. Squares depict values for individual patients with adjusted clearances due to increased intraabdominal pressure, circles depict values for study populations without adjustment of clearances. Here, each symbol represents a single concentration. The black solid line marks the line of identity. Black dotted lines indicate 1.25-fold, black dashed lines indicate 2-fold deviation. **abdom**, abdominal.

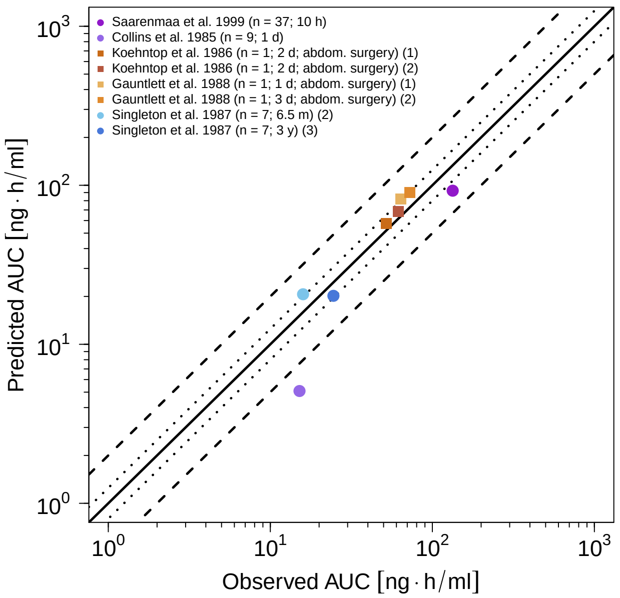

Figure S8: Predicted versus observed AUC of fentanyl for the pediatric PBPK model. Squares depict values for individual patients with adjusted clearances due to increased intraabdominal pressure, circles depict values for study populations without adjustment of clearances. Here, each symbol represents the AUC of a single concentration-time profile. The black solid line marks the line of identity. Black dotted lines indicate 1.25-fold, black dashed lines indicate 2-fold deviation. **abdom**, abdominal; **AUC**, area under the plasma concentration-time curve from the first to the last data point.

3.3 Quantitative PBPK Model Evaluation

Two quantitative performance measures, the mean relative deviations (MRD) of the predicted plasma concentrations for all observed and the respective predicted plasma concentrations and the geometric mean fold errors (GMFE) of the predicted versus observed AUC_{last} values, were calculated according to Equation S6 and Equation S7, respectively. C_{max} values were not calculated as C_{max} values of a substance administered as intravenous bolus injection or as short-term infusions are very sensitive to the timing of blood sampling.

$$MRD = 10^x \text{ with } x = \sqrt{\frac{1}{n}\sum_{i=1}^{n}(\log_{10}\hat{c}_i - \log_{10}c_i)^2} \tag{S6}$$

Here, c_i is the ith observed plasma concentration, $\hat{c}_i$ is the respective predicted plasma concentration and n equals the number of observed values. Calculated MRD values for all studies are given in Table S4.

$$GMFE = 10^x \text{ with } x = \frac{1}{n}\sum_{i=1}^{n}\left|\log_{10}\left(\frac{\hat{AUC}_i}{AUC_i}\right)\right| \tag{S7}$$

Here, AUC_i is the ith observed AUC_{last} value, $\hat{AUC}_i$ is the predicted AUC_{last} value and n equals the number of studies. The calculated GMFE values are shown in Table S5.

3.4 Mean Relative Deviation (MRD) Values of Fentanyl and Norfentanyl Plasma Concentration Predictions

Table S4: Mean relative deviation (MRD) values of fentanyl and norforfentanyl plasma concentration predictions.

Study ID	Compound	Blood Sample	Dose [µg/kg][a]	Dose [µg/h][b]	Administration	MRD	Reference
**Fentanyl iv adults**							
Bentley et al. 1982 (Adult)	Fentanyl	arterial	10.0		iv (bolus)	1.61	[40]
Bentley et al. 1982 (Eldery)	Fentanyl	arterial	10.0		iv (bolus)	1.94	[40]
Bovill and Sebel 1980	Fentanyl	venous	60.0		iv (2 min)	1.41	[41]
Christrup et al. 2008	Fentanyl	venous	1.5		iv (-)	2.56	[42]
Duthie et al. 1986 (1)	Fentanyl	venous	1.4	100.0	iv (24 h + bolus)	1.79	[43]
Duthie et al. 1986 (2)	Fentanyl	venous	1.5	100.0	iv (24 h + bolus)	2.00	[43]
Duthie et al. 1986 (3)	Fentanyl	venous	1.4	100.0	iv (24 h + bolus)	1.30	[43]
Duthie et al. 1986 (4)	Fentanyl	venous	7.2	100.0	iv (26 h + bolus)	2.37	[43]
Gourlay et al. 1989	Fentanyl	venous[c]	1		iv (1 min)	2.71	[44]
Gupta et al. 1995	Fentanyl	venous[d]		50.0	iv (48 h)	1.34	[45]
Holley and van Steennis 1988 (1)	Fentanyl	arterial	1.3	25.0	iv (loading dose + 24 h)	1.17	[46]
Holley and van Steennis 1988 (2)	Fentanyl	arterial	2.5	50.0	iv (loading dose + 24 h)	1.07	[46]
Holley and van Steennis 1988 (3)	Fentanyl	arterial	5.0	100.0	iv (loading dose + 24 h)	1.33	[46]
Holley and van Steennis 1988 (4)	Fentanyl	arterial	6.5	125.0	iv (loading dose + 24 h)	1.22	[46]
Lim et al. 2012	Fentanyl	venous	1.5		iv (5 min)	2.62	[47]
MacLeod et al. 2012	Fentanyl	arterial	0.3		iv (5 sec)	1.49	[48]
McClain and Hug 1980	Fentanyl	arterial	6.4		iv (1.5 min)	1.77	[9]
Saari et al. 2008	Fentanyl	venous	5.0		iv (2 min)	1.49	[7]
Saari et al. 2008	Norfentanyl	venous	5.0		iv (2 min)	1.87	[7]
Saari et al. 2008 (DDI)	Fentanyl	venous	5.0		iv (2 min)	1.51	[7]
Saari et al. 2008 (DDI)	Norfentanyl	venous	5.0		iv (2 min)	2.40	[7]
Singleton et al. 1987 (1)	Fentanyl	arterial	20.7		iv (2 min)	1.65	[17]
Stoeckel et al. 1982	Fentanyl	venous	7.6		iv (bolus)	2.00	[49]
Streisand et al. 1991	Fentanyl	arterial	15.0		iv (8 min)	1.87	[50]
Varvel et al. 1989	Fentanyl	arterial	11.4		iv (5 min)	1.51	[51]
Varvel et al. 1989	Fentanyl	venous	11.4		iv (5 min)	1.29	[51]

[a] dose of bolus injection or short-infusion
[b] dose of long-term infusion
[c] venous blood samples from a central venous catheter
[d] sample information was not specified, venous blood samples were assumed
DDI: drug-drug-interaction, **iv**: intravenous, **MRD**: mean relative deviation

Table S4: Mean relative deviation (MRD) values of fentanyl and norforfentanyl plasma concentration predictions. *(continued)*

Study ID	Compound	Blood Sample	Dose [µg/kg][a]	Dose [µg/h][b]	Administration	MRD	Reference
Ziesenitz et al. 2015	Fentanyl	venous	5.0		iv (10 min)	1.72	[8]
Ziesenitz et al. 2015	Norfentanyl	venous	5.0		iv (10 min)	1.12	[8]
**MRD**						**1.77 (1.07–2.71)**	
						**22/28 with MRD ≤ 2**	
**Fentanyl iv children**							
Collins et al. 1985	Fentanyl	arterial	30.0		iv (1 min)	3.16	[52]
Gauntlett et al. 1988 (1)	Fentanyl	arterial	52.5		iv (2 min)	2.43	[23]
Gauntlett et al. 1988 (2)	Fentanyl	arterial	56.5		iv (2 min)	2.25	[23]
Koehntop et al. 1986 (1)	Fentanyl	arterial	25.0		iv (1–3 min)	2.62	[24]
Koehntop et al. 1986 (2)	Fentanyl	arterial	50.0		iv (1–3 min)	1.71	[24]
Saarenmaa et al. 2000	Fentanyl	arterial	10.5	1.5	iv (1 h + 58 h)	1.79	[18]
Singleton et al. 1987 (2)	Fentanyl	arterial	31.2		iv (2 min)	1.53	[17]
Singleton et al. 1987 (3)	Fentanyl	venous	30.8		iv (2 min)	1.64	[17]
**MRD**						**2.04 (1.53–3.16)**	
						**4/8 with MRD ≤ 2**	

[a] dose of bolus injection or short-infusion
[b] dose of long-term infusion
[c] venous blood samples from a central venous catheter
[d] sample information was not specified, venous blood samples were assumed
DDI: drug-drug-interaction, **iv**: intravenous, **MRD**: mean relative deviation

3.5 Geometric Mean Fold Error (GMFE) of AUC_{last} Predictions

Table S5: Predicted and observed AUC_{last} values of fentanyl and norfentanyl plasma concentrations.

						AUC_{last}			
Study ID	Compound	Blood Sample	Dose [µg/kg][a]	Dose [µg/h][b]	Administration	Pred [ng·h/ml]	Obs [ng·h/ml]	Pred/Obs	Reference
**Fentanyl iv adults**									
Bentley et al. 1982 (Adult)	Fentanyl	arterial	10.0		iv (bolus)	7.08	8.58	0.83	[40]
Bentley et al. 1982 (Eldery)	Fentanyl	arterial	10.0		iv (bolus)	8.55	14.47	0.59	[40]
Bovill and Sebel 1980	Fentanyl	venous	60.0		iv (2 min)	40.76	34.67	1.18	[41]
Christrup et al. 2008	Fentanyl	venous	1.5		iv (-)	0.93	0.81	1.15	[42]
Duthie et al. 1986 (1)	Fentanyl	venous	1.4	100.0	iv (24 h + bolus)	45.21	42.71	1.06	[43]
Duthie et al. 1986 (2)	Fentanyl	venous	1.5	100.0	iv (24 h + bolus)	47.42	50.60	0.94	[43]
Duthie et al. 1986 (3)	Fentanyl	venous	1.4	100.0	iv (24 h + bolus)	44.10	41.61	1.06	[43]
Duthie et al. 1986 (4)	Fentanyl	venous	7.2	100.0	iv (26 h + bolus)	55.72	69.00	0.81	[43]
Gourlay et al. 1989	Fentanyl	venous[c]	1		iv (1 min)	0.36	0.16	2.30	[44]
Gupta et al. 1995	Fentanyl	venous[d]		50.0	iv (48 h)	51.12	64.32	0.79	[45]
Holley and van Steennis 1988 (1)	Fentanyl	arterial	1.3	25.0	iv (loading dose + 24 h)	10.18	11.48	0.89	[46]
Holley and van Steennis 1988 (2)	Fentanyl	arterial	2.5	50.0	iv (loading dose + 24 h)	19.31	19.87	0.97	[46]
Holley and van Steennis 1988 (3)	Fentanyl	arterial	5.0	100.0	iv (loading dose + 24 h)	40.55	31.57	1.28	[46]
Holley and van Steennis 1988 (4)	Fentanyl	arterial	6.5	125.0	iv (loading dose + 24 h)	50.81	42.43	1.20	[46]
Lim et al. 2012	Fentanyl	venous	1.5		iv (5 min)	2.19	1.67	1.31	[47]
MacLeod et al. 2012	Fentanyl	arterial	0.3		iv (5 sec)	0.36	0.31	1.14	[48]
McClain and Hug 1980	Fentanyl	arterial	6.4		iv (1.5 min)	6.27	6.58	0.95	[9]
Saari et al. 2008	Fentanyl	venous	5.0		iv (2 min)	3.86	4.71	0.82	[7]
Saari et al. 2008	Norfentanyl	venous	5.0		iv (2 min)	2.13	1.31	1.62	[7]
Saari et al. 2008 (DDI)	Fentanyl	venous	5.0		iv (2 min)	4.71	6.25	0.75	[7]
Saari et al. 2008 (DDI)	Norfentanyl	venous	5.0		iv (2 min)	0.05	0.11	0.44	[7]
Singleton et al. 1987 (1)	Fentanyl	arterial	20.7		iv (2 min)	18.70	18.66	1.00	[17]
Stoeckel et al. 1982	Fentanyl	venous	7.6		iv (bolus)	4.89	8.06	0.61	[49]
Streisand et al. 1991	Fentanyl	arterial	15.0		iv (8 min)	13.99	25.51	0.55	[50]
Varvel et al. 1989	Fentanyl	arterial	11.4		iv (5 min)	5.99	8.49	0.71	[51]
Varvel et al. 1989	Fentanyl	venous	11.4		iv (5 min)	3.05	2.78	1.10	[51]
Ziesenitz et al. 2015	Fentanyl	venous	5.0		iv (10 min)	6.41	4.22	1.52	[8]
Ziesenitz et al. 2015	Norfentanyl	venous	5.0		iv (10 min)	2.54	2.57	0.99	[8]
**GMFE**								**1.30 (1.00–2.30)**	
								**26/28 with GMFE ≤ 2**	
**Fentanyl iv children**									
Collins et al. 1985	Fentanyl	arterial	30.0		iv (1 min)	5.09	15.14	0.34	[52]
Gauntlett et al. 1988 (1)	Fentanyl	arterial	52.5		iv (2 min)	81.92	63.85	1.28	[23]
Gauntlett et al. 1988 (2)	Fentanyl	arterial	56.5		iv (2 min)	90.19	72.67	1.24	[23]
Koehntop et al. 1986 (1)	Fentanyl	arterial	25.0		iv (1–3 min)	57.43	51.80	1.11	[24]
Koehntop et al. 1986 (2)	Fentanyl	arterial	50.0		iv (1–3 min)	68.19	61.67	1.11	[24]
Saarenmaa et al. 2000	Fentanyl	arterial	10.5	1.5	iv (1 h + 58 h)	92.50	133.53	0.69	[18]
Singleton et al. 1987 (2)	Fentanyl	arterial	31.2		iv (2 min)	20.65	15.93	1.30	[17]
Singleton et al. 1987 (3)	Fentanyl	venous[a]	30.8		iv (2 min)	20.18	24.51	0.82	[17]
**GMFE**								**1.38 (1.11–2.98)**	
								**7/8 with GMFE ≤ 2**	

[a] dose of bolus injection or short-infusion
[b] dose of long-term infusion
[c] venous blood samples from a central venous catheter
[d] sample information was not specified, venous blood samples were assumed
DDI: drug-drug-interaction, **GMFE**: geometric mean fold error, **iv**: intravenous, **Obs**: observed, **Pred**: predicted

3.6 Fentanyl and Norfentanyl PBPK Model Sensitivity Analysis

A sensitivity analysis of the developed PBPK models (adults and pediatrics) to single parameter changes was performed (local sensitivity analysis). It needs to be noted, that sensitivity to parameters regarding the metabolite norfentanyl was not investigated for the pediatric models as norfentanyl plasma concentration measurements were only available in clinical studies with adults. In case of full-term neonates, sensitivity was examined for model parameters (1) with metabolic clearance adaption due to increased intraabdominal pressure (see Section 1.2) and (2) without metabolic clearance adaption. Sensitivities of the PBPK models were calculated as the relative changes of the predicted area under the plasma concentration-time curve extrapolated to infinity ($\mathrm{AUC_{inf}}$) of fentanyl and norfentanyl, respectively, to the relative variation of model input parameters in a short infusion scenario (20.7 µg/kg fentanyl administered over two minutes [17]). Parameters, optimized as well as parameters fixed to literature values, were included into the analysis if they had significant impact in former models (e.g. glomerular filtration rate fraction), if they could have a decisive influence due to calculation methods used in the model (e.g. lipophilicity) and/or if they have been optimized. The analyses were performed using a relative perturbation of parameters of 10%. Model sensitivity to a parameter was calculated as follows:

$$S = \frac{\Delta AUC_{inf}}{\Delta p} \cdot \frac{p}{AUC_{inf}} \tag{S8}$$

where S is the sensitivity of the AUC_{inf} to the examined model parameter, ΔAUC_{inf} is the change of the AUC_{inf}, AUC_{inf} is the simulated AUC_{inf} with the original parameter value, p is the original model parameter value and Δp is the change of the model parameter value. A sensitivity value of +1.0 signifies that a 10% increase of the examined parameter causes a 10 % increase of the simulated $\mathrm{AUC_{inf}}$.

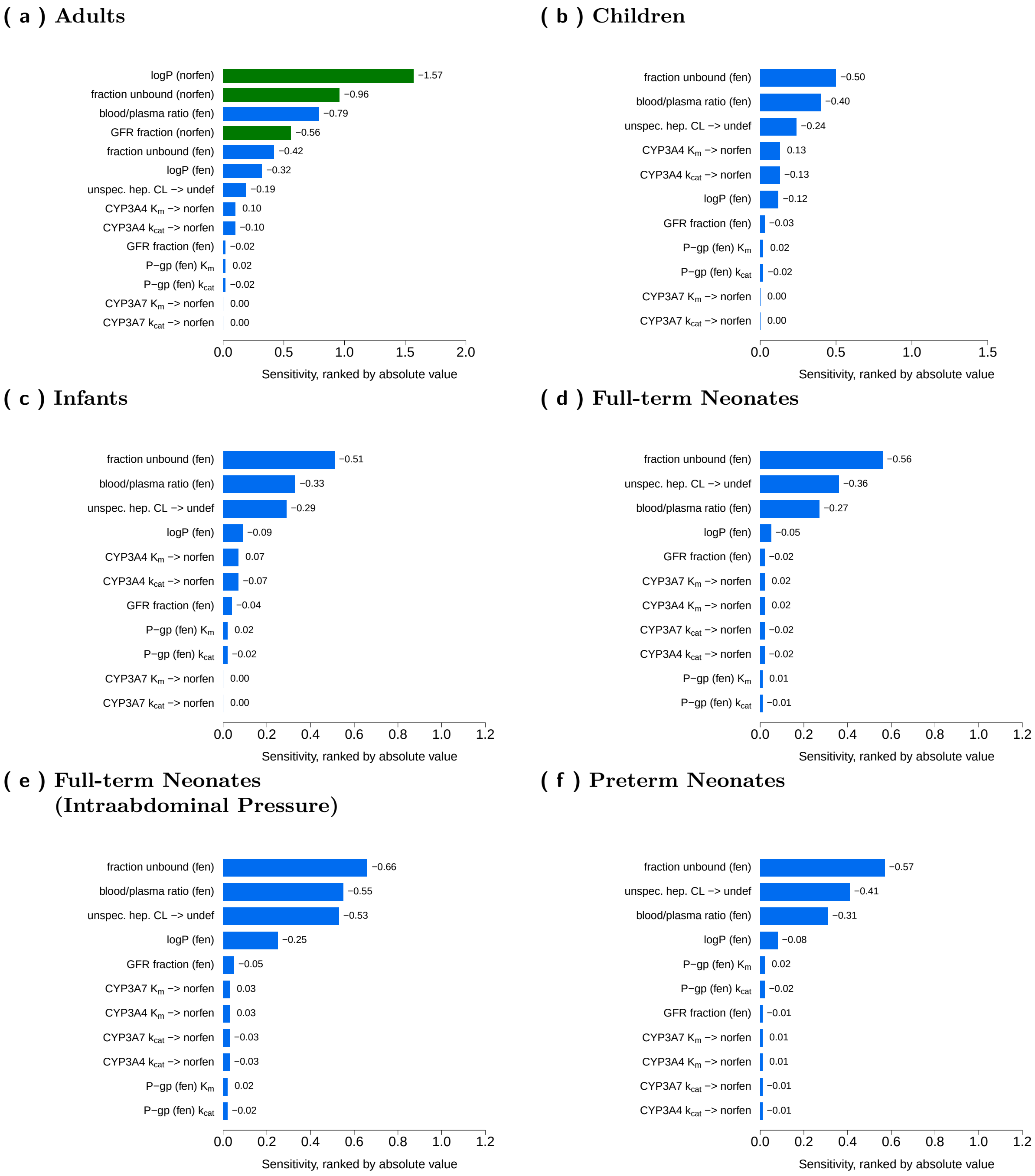

Figure S9: Sensitivity analyses of the fentanyl PBPK model in different populations. Sensitivity of the model to single parameters, calculated as change of the simulated AUC$_{inf}$ of fentanyl and norfentanyl, respectively, following a short infusion scenario ($20.7\,\mu g/kg$ of fentanyl administered over two minutes [17]). **AUC$_{inf}$**: area under the plasma concentration-time curve extrapolated to infinity, **CYP**: Cytochrome P450, **fen**: fentanyl, **GFR**: glomerular filtration rate, **k_{cat}**: catalytic rate constant, **K_m**: Michaelis-Menten constant, **norfen**: norfentanyl, **P-gp**: P-glycoprotein, **undef**: undefined metabolite, **unspec. hep. CL**: unspecific hepatic clearance.

References

[1] Maharaj AR, Barrett JS, Edginton AN (2013) A workflow example of PBPK modeling to support pediatric research and development: case study with lorazepam. The AAPS journal 15(2):455–64

[2] Maharaj AR, Edginton AN (2014) Physiologically Based Pharmacokinetic Modeling and Simulation in Pediatric Drug Development. CPT: Pharmacometrics & Systems Pharmacology 3(11):1–13

[3] Leong R, Vieira MLT, Zhao P, Mulugeta Y, Lee CS, Huang SM, Burckart GJ (2012) Regulatory experience with physiologically based pharmacokinetic modeling for pediatric drug trials. Clinical pharmacology and therapeutics 91(5):926–31

[4] Ince I, Solodenko J, Frechen S, Dallmann A, Niederalt C, Schlender J, Burghaus R, Lippert J, Willmann S (2019) Predictive Pediatric Modeling and Simulation Using Ontogeny Information. The Journal of Clinical Pharmacology 59(S1):S95–S103

[5] Kovar L, Schräpel C, Selzer D, Kohl Y, Bals R, Schwab M, Lehr T (2020) Physiologically-Based Pharmacokinetic (PBPK) Modeling of Buprenorphine in Adults, Children and Preterm Neonates. Pharmaceutics 12(6):578

[6] Feierman DE, Lasker JM (1996) Metabolism of fentanyl, a synthetic opioid analgesic, by human liver microsomes. Role of CYP3A4. Drug metabolism and disposition: the biological fate of chemicals 24(9):932–9

[7] Saari TI, Laine K, Neuvonen M, Neuvonen PJ, Olkkola KT (2008) Effect of voriconazole and fluconazole on the pharmacokinetics of intravenous fentanyl. European Journal of Clinical Pharmacology 64(1):25–30

[8] Ziesenitz VC, König SK, Mahlke NS, Skopp G, Haefeli WE, Mikus G (2015) Pharmacokinetic interaction of intravenous fentanyl with ketoconazole. Journal of Clinical Pharmacology 55(6):708–717

[9] McClain DA, Hug CC (1980) Intravenous fentanyl kinetics. Clinical Pharmacology and Therapeutics 28(1):106–114

[10] Lötsch J, Walter C, Parnham MJ, Oertel BG, Geisslinger G (2013) Pharmacokinetics of Non-Intravenous Formulations of Fentanyl. Clinical Pharmacokinetics 52(1):23–36

[11] Williams JA, Ring BJ, Cantrell VE, Jones DR, Eckstein J, Ruterbories K, Hamman MA, Hall SD, Wrighton SA (2002) Comparative Metabolic Capabilities of CYP3A4, CYP3A5, and CYP3A7. Drug Metabolism and Disposition 30(8):883–891

[12] Niwa T, Okamoto A, Narita K, Toyota M, Kato K, Kobayashi K, Sasaki S (2020) Comparison of steroid hormone hydroxylation mediated by cytochrome P450 3A subfamilies. Archives of Biochemistry and Biophysics 682:108283

[13] Yu C, Yuan M, Yang H, Zhuang X, Li H (2018) P-glycoprotein on blood-brain barrier plays a vital role in fentanyl brain exposure and respiratory toxicity in rats. Toxicological Sciences 164(1):353–362

[14] Henthorn TK, Liu Y, Mahapatro M, Ng KY (1999) Active transport of fentanyl by the blood-brain barrier. Journal of Pharmacology and Experimental Therapeutics 289(2):1084–1089

[15] Thompson SJ, Koszdin K, Bernards CM (2000) Opiate-induced analgesia is increased and prolonged in mice lacking P- glycoprotein. Anesthesiology 92(5):1392–1399

[16] Kharasch ED, Hoffer C, Altuntas TG, Whittington D (2004) Quinidine as a Probe for the Role of P-Glycoprotein in the Intestinal Absorption and Clinical Effects of Fentanyl. Journal of Clinical Pharmacology 44(3):224–233

[17] Singleton Ma, Rosen JI, Fisher DM (1987) Plasma concentrations of fentanyl in infants, children and adults. Canadian Journal of Anaesthesia 34(2):152–155

[18] Saarenmaa E, Neuvonen PJ, Fellman V (2000) Gestational age and birth weight effects on plasma clearance of fentanyl in newborn infants. Journal of Pediatrics 136(6):0767–0770

[19] Rodgers T, Rowland M (2006) Physiologically-based Pharmacokinetic Modeling 2: Predicting the tissue distribution of acids, very weak bases, neutrals and zwitterions. Journal of Pharmaceutical Sciences 95:1238–1257

[20] Rodgers T, Leahy D, Rowland M (2005) Physiologically based pharmacokinetic modeling 1: Predicting the tissue distribution of moderate-to-strong bases. Journal of Pharmaceutical Sciences 94:1259–1276

[21] Rodgers T, Rowland M (2007) Mechanistic approaches to volume of distribution predictions: understanding the processes. Pharmaceutical research 24(5):918–33

[22] Schmitt W (2008) General approach for the calculation of tissue to plasma partition coefficients. Toxicology in vitro : an international journal published in association with BIBRA 22(2):457–67

[23] Gauntlett IS, Fisher DM, Hertzka RE, Kuhis E, Spellman MJ, Rudolph C (1988) Pharmacokinetics of Fentanyl in Neonatal Humans and Lambs. Anesthesiology 69(5):683–687

[24] Koehntop DE, Rodman JH, Brundage DM, Hegland MG, Buckley JJ (1986) Pharmacokinetics of fentanyl in neonates. Anesthesia and Analgesia 65(3):227–232

[25] Willmann S, Höhn K, Edginton A, Sevestre M, Solodenko J, Weiss W, Lippert J, Schmitt W (2007) Development of a physiology-based whole-body population model for assessing the influence of individual variability on the pharmacokinetics of drugs. Journal of Pharmacokinetics and Pharmacodynamics 34(3):401–431

[26] Stader F, Penny MA, Siccardi M, Marzolini C (2019) A Comprehensive Framework for Physiologically-Based Pharmacokinetic Modeling in Matlab. CPT: Pharmacometrics & Systems Pharmacology 8(7):psp4.12399

[27] Valentin J (2002) Basic anatomical and physiological data for use in radiological protection: reference values. Annals of the ICRP 32(3-4):1–277

[28] National Center for Health Statistics (1997) Third National Health and Nutrition Examination Survey (NHANES III). Tech. rep., Hyattsville, MD 20782

[29] Nishimura M, Yaguti H, Yoshitsugu H, Naito S, Satoh T (2003) Tissue distribution of mRNA expression of human cytochrome P450 isoforms assessed by high-sensitivity real-time reverse transcription PCR. Journal of the Pharmaceutical Society of Japan 123(5):369–75

[30] Nishimura M, Naito S (2005) Tissue-specific mRNA Expression Profiles of Human ATP-binding Cassette and Solute Carrier Transporter Superfamilies. Drug Metabolism and Pharmacokinetics 20(6):452–477

[31] Nishimura M, Naito S (2006) Tissue-Specific mRNA Expression Profiles of Human Phase I Metabolizing Enzymes Except for Cytochrome P450 and Phase II Metabolizing Enzymes. Drug Metabolism and Pharmacokinetics 21(5):357–374

[32] Rodrigues AD (1999) Integrated cytochrome P450 reaction phenotyping: attempting to bridge the gap between cDNA-expressed cytochromes P450 and native human liver microsomes. Biochemical pharmacology 57(5):465–80

[33] Open Systems Pharmacology Suite Community (2018) PK-Sim® Ontogeny Database Documentation, Version 7.3. https://github.com/Open-Systems-Pharmacology/OSPSuite.Documentation/blob/master/PK-SimOntogenyDatabaseVersion7.3.pdf, accessed: 2020-03-25

[34] Stevens JC, Hines RN, Gu C, Koukouritaki SB, Manro JR, Tandler PJ, Zaya MJ (2003) Developmental Expression of the Major Human Hepatic CYP3A Enzymes. Journal of Pharmacology and Experimental Therapeutics 307(2):573–582

[35] Prasad B, Evers R, Gupta A, Hop CECA, Salphati L, Shukla S, Ambudkar SV, Unadkat JD (2014) Interindividual Variability in Hepatic Organic Anion-Transporting Polypeptides and P-Glycoprotein (ABCB1) Protein Expression: Quantification by Liquid Chromatography Tandem Mass Spectroscopy and Influence of Genotype, Age, and Sex. Drug Metabolism and Disposition 42(1):78–88

[36] Prasad B, Gaedigk A, Vrana M, Gaedigk R, Leeder J, Salphati L, Chu X, Xiao G, Hop C, Evers R, Gan L, Unadkat J (2016) Ontogeny of Hepatic Drug Transporters as Quantified by LC-MS/MS Proteomics. Clinical Pharmacology & Therapeutics 100(4):362–370

[37] Meyer M, Schneckener S, Ludewig B, Kuepfer L, Lippert J (2012) Using Expression Data for Quantification of Active Processes in PBPK Modeling. Drug Metabolism and Disposition 40(5):892–901

[38] Hanke N, Frechen S, Moj D, Britz H, Eissing T, Wendl T, Lehr T (2018) PBPK Models for CYP3A4 and P-gp DDI Prediction: A Modeling Network of Rifampicin, Itraconazole, Clarithromycin, Midazolam, Alfentanil, and Digoxin. CPT: Pharmacometrics and Systems Pharmacology 7(10):647–659

[39] Li X, Frechen S, Moj D, Lehr T, Taubert M, hsuan Hsin C, Mikus G, Neuvonen PJ, Olkkola KT, Saari TI, Fuhr U (2019) A Physiologically Based Pharmacokinetic Model of Voriconazole Integrating Time-Dependent Inhibition of CYP3A4, Genetic Polymorphisms of CYP2C19 and Predictions of Drug–Drug Interactions. Clinical Pharmacokinetics (0123456789)

[40] Bentley JB, Borel JD, Nenad RE, Gillespie TJ (1982) Age and Fentanyl Pharmacokinetics. Anesthesia & Analgesia 61(12):968–971

[41] Bovill JG, Sebel PS (1980) Pharmacokinetics of high-dose fentanyl: A study in patients undergoing cardiac surgery. British Journal of Anaesthesia 52(8):795–801

[42] Christrup L, Foster D, Popper L, Troen T, Upton R (2008) Pharmacokinetics, efficacy, and tolerability of fentanyl following intranasal versus intravenous administration in adults undergoing third-molar extraction: A randomized, double-blind, double-dummy, two-way, crossover study. Clinical Therapeutics 30(3):469–481

[43] Duthie D, Mclaren A, Nimmo W (1986) Pharmacokinetics Of Fentanyl During Constant Rate I.V. Infusion For The Relief Of Pain After Surgery. British Journal of Anaesthesia 58(9):950–956

[44] Gourlay GK, Kowalski SR, Plummer JL, Cherry DA, Gaukroger P, Cousins MJ (1989) The transdermal administration of fentanyl in the treatment of postoperative pain: pharmacokinetics and pharmacodynamic effects. Pain 37(2):193–202

[45] Gupta SK, Southam MA, Hwang SS (1995) Evaluation of Diurnal Variation in Fentanyl Clearance. The Journal of Clinical Pharmacology 35(2):159–162

[46] Holley FO, Van Steennis C (1988) Postoperative analgesia with fentanyl: Pharmacokinetics and pharmacodynamics of constant-rate I.V. and transdermal delivery. British Journal of Anaesthesia 60(6):608–613

[47] Lim CBS, Schug SA, Sunderland VB, Paech MJ, Liu Y (2012) A Phase I Pharmacokinetic and Bioavailability Study of a Sublingual Fentanyl Wafer in Healthy Volunteers. Anesthesia & Analgesia 115(3):1

[48] MacLeod DB, Habib AS, Ikeda K, Spyker DA, Cassella JV, Ho KY, Gan TJ (2012) Inhaled Fentanyl Aerosol in Healthy Volunteers. Anesthesia & Analgesia 115(5):1071–1077

[49] Stoeckel H, Schüttler J, Magnussen H, Hengstmann J (1982) Plasma Fentanyl Concentrations And The Occurrence Of Respiratory Depression In Volunteers. British Journal of Anaesthesia 54(10):1087–1095

[50] Streisand JB, Varvel JR, Stanski DR, Maire LL, Ashburn MA, Hague BI, Tarver SD, Stanley TH (1991) Absorption and Bioavailability of Oral Transmucosal Fentanyl Citrate. Anesthesiology 75(2):223–229

[51] Varvel JR, Shafer SL, Hwang SS, Coen PA, Stanski DR (1989) Absorption Characteristics of Transdermally Administered Fentanyl. Anesthesiology 70(6):928–934

[52] Collins C, Koren G, Crean P, Klein J, Roy WL, MacLeod SM (1985) Fentanyl pharmacokinetics and hemodynamic effects in preterm infants during ligation of patent ductus arteriosus. Anesthesia and Analgesia 64(11):1078–1080
